# Chemical and Physical Architecture of Macromolecular Gels for Fracturing Fluid Applications in the Oil and Gas Industry; Current Status, Challenges, and Prospects

**DOI:** 10.3390/gels10050338

**Published:** 2024-05-16

**Authors:** Majad Khan

**Affiliations:** 1 Department of Chemistry, King Fahd University of Petroleum & Minerals (KFUPM), Dhahran 31261, Saudi Arabia; majad.khan@kfupm.edu.sa; Tel.: +966-0138601671; 2 Interdisciplinary Research Center for Hydrogen Technologies and Energy Storage (IRC-HTCM), King Fahd University of Petroleum & Minerals (KFUPM), Dhahran 31261, Saudi Arabia; 3 Interdisciplinary Research Center for Refining and Advanced Chemicals (IRC-CRAC), King Fahd University of Petroleum & Minerals (KFUPM), Dhahran 31261, Saudi Arabia

**Keywords:** fracturing fluids, macromolecules, gels, natural polymers, synthetic polymers, hyperbranched polymers, viscosity, shear thinning, crosslinked polymers

## Abstract

Hydraulic fracturing is vital in recovering hydrocarbons from oil and gas reservoirs. It involves injecting a fluid under high pressure into reservoir rock. A significant part of fracturing fluids is the addition of polymers that become gels or gel-like under reservoir conditions. Polymers are employed as viscosifiers and friction reducers to provide proppants in fracturing fluids as a transport medium. There are numerous systems for fracturing fluids based on macromolecules. The employment of natural and man-made linear polymers, and also, to a lesser extent, synthetic hyperbranched polymers, as additives in fracturing fluids in the past one to two decades has shown great promise in enhancing the stability of fracturing fluids under various challenging reservoir conditions. Modern innovations demonstrate the importance of developing chemical structures and properties to improve performance. Key challenges include maintaining viscosity under reservoir conditions and achieving suitable shear-thinning behavior. The physical architecture of macromolecules and novel crosslinking processes are essential in addressing these issues. The effect of macromolecule interactions on reservoir conditions is very critical in regard to efficient fluid qualities and successful fracturing operations. In future, there is the potential for ongoing studies to produce specialized macromolecular solutions for increased efficiency and sustainability in oil and gas applications.

## 1. Introduction

Fluids composed of various additives are injected at high pressure into the reservoir rock to give rise to fractures within the rock. This describes the process of hydraulic fracturing, which is a vital method in the recovery of hydrocarbons from such reservoirs [1,2,3]. This hydraulic fracturing process results in improved productivity of petroleum from crude oil reservoirs. An ideal fracturing fluid should have appropriate characteristics, such as efficiently supporting proppant transportation and being compatible with formation rock and other reservoir materials, environmental benignity, and cost-effectiveness. Proppant is introduced into fractures created by hydraulic fracturing using fracturing fluid as a transport medium, which results in effective conductivity to encourage the movement of oil and/or gas efficiently from formation rock [4]. Proppants are essential for optimal hydraulic fracturing to improve oil and gas extraction, particularly for old wells and poor permeability reservoirs. Proppant is a solid particle with spherical and circularity, strength, corrosion resistance, and stability that can prevent hydraulic cracks from re-closing and keep oil and gas flowing smoothly, thus increasing production. In the hydraulic fracturing process, the proppant’s shape, strength, acid solubility, and turbidity may affect the integrity of newly created cracks, influencing the efficiency of oil and gas flow from the well [5]. Oil-based fracturing fluids are associated with environmental and safety concerns. While the water-based fluids supported with macromolecular additives can increase viscosity and help transport proppants, as a result, these are considered attractive substitutes to oil-based fracturing fluids. Slick water is another popular type of fracturing fluid method, and it involves pumping water at very high rates (>60 bpm) followed by injection of a crosslinked material (gel) to transport proppant. Although this technique has the advantage of cost-effectiveness, it suffers from multiple disadvantages, such as difficulty in modeling the fracture network because of its complex geometry, reduced proppant transport, and insignificant fracture widths due to injection of low-viscosity fluids.

As in the past, most fracturing fluids are primarily composed of polymers. Polymers are large molecules (macromolecules) composed of smaller subunits known as monomers. Broadly speaking, a majority of natural and man-made polymers are linear in terms of physical architecture. Macromolecular chemistry is at the forefront of providing enhanced formulations for fracturing fluids used in hydraulic fracturing, commonly known as fracking, in the oil and gas industry. Fracturing fluids are used to enable fractures in deep underground reservoir rock, allowing oil and gas to be extracted from previously inaccessible or difficult-to-recover sources. Fracturing fluids must have appropriate viscosity parameters, such as behaving like gels once positioned inside reservoir/fractures to efficiently transport proppants into the cracks formed and keep them open after the fracturing process is complete.

Natural polymers are derived from sustainable sources such as plants, seeds, fruits, and animals. These include cellulose, starch, guar, and xanthan gum, which are widely used in various applications, including hydraulic fracturing. Cellulose is a polysaccharide found in plant cell walls that is employed as a thickening agent. It can help reduce fluid loss in fracturing fluids due to its large molecular weight and water-retention capabilities. Linear or crosslinked guar gums tend to be high-molecular-weight biopolymers that bear mannose and galactose sugars, which are commonly used fluids in fracturing operations. However, guar gums suffer from the limited supply of guar produced from beans and intolerance to elevated temperatures (up to 210 °F) [6]. Two of the most common biopolymers are starch and cellulose, which are readily available, environmentally friendly, and biodegradable, making them an ideal polymeric additive for enhanced oil recovery. Moreover, they can overcome difficult reservoir conditions due to their robust chemical structure and high molecular weights. Xanthan gum is another popular biopolymer processed for typical polymer flooding applications. Before its use in oil recovery, xanthan gum was utilized in a few industries, such as food and sweets. Several studies have found that natural polymers significantly improve oil recovery efficiencies [7].

Man-made polymers, such as hydrolyzed polyacrylamide (HPAM) and polyacrylamide (PAM), have been employed as viscosity-enhancing additives and biopolymers during common flooding applications. Although synthetic polymers often have uniform basic structures, their use in the petroleum recovery process is restricted due to cost issues resulting from the employment of difficult synthetic methods and posing potential environmental hazards [8]. In the field of enhanced oil recovery, amongst the various polymers that are utilized as additives, HPAM is a popular choice due to its high molecular weight, adaptable characteristics, and affordable manufacturing costs. However, such manufactured polymers are vulnerable to harsh reservoir conditions that can cause a significant reduction in viscosity [9].

Until now, all the polymers employed as fracturing fluids or additives in fracturing fluids have primarily been linear biopolymers or synthetic linear polymers. Recently, researchers have started to investigate highly branched polymers (hyperbranched polymers) as fracturing fluid additives [10]. Hyperbranched polymers in fracturing fluids have higher viscosity at lower concentrations than linear polymers, allowing for better proppant suspension and transport. Their branching structure makes them more resistant to mechanical degradation under high shear conditions, leading to sustained fracture conductivity. Moreover, hyperbranched polymers should possess improved fluid flow characteristics, which help to reduce loss due to friction in the wellbore and increase pumping efficiency. This enhanced performance will enable hyperbranched polymers to increase efficiency in hydraulic fracturing operations, which may lead to increased well productivity and optimal resource recovery.

Consequently, there is an urgent need to explore this area of polymer chemistry to understand how these macromolecules’ chemical and physical architectures can enhance the applications of fracturing fluids in oil and gas recovery. This review aims to comprehensively investigate and clarify the complex chemistry and physical architecture of macromolecules used in fracturing fluid applications in the oil and gas industry. Moreover, the objective of this review is to provide a detailed understanding of the advantages and drawbacks of these macromolecular solutions by evaluating their current state, as well as to provide a comprehensive and forward-looking view of the changing environment of macromolecular solutions in the oil and gas industry, covering both challenges and future potential.

## 2. Macromolecular Chemistry in Fracturing Fluids

**Figure 1:**
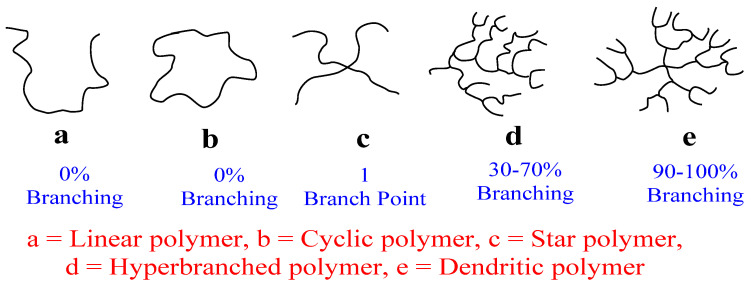
Types of physical architecture in macromolecules and approximate degree of branching for a given type of physical architecture in macromolecules.

When one considers the subject of synthetic polymer chemistry over the past three decades or so, dendritic macromolecules have received more than their fair share of attention from researchers and industry. Why is this so? Could they pose a synthetic challenge in obtaining a perfectly dendritic macromolecule produced in a “one-pot” procedure? On the other hand, do dendritic macromolecules have interesting physical and chemical properties because of their branched architecture? Dendrimers and hyperbranched macromolecules are examples of polymers belonging to the branched subfamily. Both types are examples of dendritic macromolecules; however, ideal dendrimers are supposed to have a degree of branching value of 1 (0.9–1.0), whereas hyperbranched polymers can have a varied degree of branching, which most often ranges from 0.3 to 0.7. In contrast, linear polymers possess zero degree of branching, and the physical architecture with approximate values of degree of branching of the different types of macromolecules can be seen in Figure 1 below.

Hyperbranched polymers may or may not possess a central core, and they are never perfectly symmetrical in their bonding architecture. Hyperbranched polymers are similar to dendrimers in their bonding architecture. They may have a central core and tend to have a higher density of surface functional groups than their linear analogs; however, unlike dendrimers, they tend to have polydisperse molecular weights, and, in this regard, the resemblance is more akin to linear polymers. Essentially, hyperbranched polymers are intermediates of dendrimers on one extreme and linear polymers on the other.

Due to the geometry of dendrimers and hyperbranched polymers, as well as the abundance of functional groups on the periphery of these macromolecules, it has been speculated that they could potentially make excellent crosslinking agents for currently used guar gum-based polymers [11]. This higher crosslinking will help reduce the amount of guar gum polymers employed as fracturing fluids and enable the engineers to have better control over the gelation/degradation of such fracturing fluids. In addition, it is possible to alter the surface functional groups with relevant chemistry to prepare synthetic versions of fracturing fluids that enable excellent control over gelation while using less polymer to make cost-effective new-generation fracturing fluids [12].

Dendritic macromolecules have captivated scientists’ minds for several decades now. The main reason for this interest is not simply due to the aesthetically pleasing architecture of the macromolecule concerned, but also the promise of interesting physical properties [13] and possible applications. It is well known that chemical functionality and the structure of the bonding units in a given macromolecule impact its physical properties [14]; therefore, the presence or absence of branching will strongly influence its physical, chemical, and mechanical properties. Regarding the synthesis of dendrimers, synthetic chemists primarily utilize the following two synthetic techniques to prepare dendrimers: Divergent and convergent routes. The main advantage of the divergent and convergent routes is that one can produce dendrimers of higher molecular weights. In the convergent growth route, bringing bulky dendrons together to a central core unit for attachment can be challenging due to steric hindrance. This challenge may be insurmountable with very large dendrons. As a result, this would most probably make it difficult to prepare dendrimers of high molecular weights via the conventional convergent growth route.

The divergent growth route also has its limitations, as noted by Tomalia [15], in that the resultant high molecular weight dendrimers formed by this route may possess less symmetry than expected. Through time-sequenced propagation techniques, the researchers successfully synthesized a novel class of topological macromolecules, termed starburst polymers, to achieve controlled molecular weight building, branching versatile dendrimer design with extraordinary symmetry, and maximized terminal functionality density. This is because, to ensure complete symmetry, all the amines in a given generation must react, which can be difficult for a high-generation dendrimer. Large amounts of highly pure reagents must be employed at every stage of dendrimer growth to reduce side reactions and increase yields. As a result, this can make purification difficult and expensive. Therefore, it is of the utmost importance that every step gives rise to a very pure product with a divergent growth route, as unwanted side reactions hinder the formation of perfect dendrimers.

As demonstrated by numerous studies over the recent past, the synthesis of the perfect dendrimer involves tedious chemistry coupled with many synthetic steps. This inevitably raises their cost of production, making them expensive additives. This primary factor prevents them from becoming widely available. Conversely, hyperbranched polymers are relatively more straightforward to synthesize and are cheaper to manufacture, making them appropriate candidates for industrial applications. The main challenge in preparing hyperbranched macromolecules is to synthesize them in a controlled way to mimic dendritic properties as much as possible without using laborious multistep synthesis protocols. The low degree of chain entanglements in hyperbranched polymers means they will possess poor mechanical properties, making them brittle and more likely to be amorphous.

Consequently, by combining this low viscosity property of these polymers with a careful selection of chemical functionalities, one can prepare hyperbranched polymers as additives that can be fine-tuned to dissolve and operate under certain reservoir conditions. As macromolecules have been thoroughly tested for their employment in enhanced oil recovery processes, researchers have been exploring the use of polymers to improve the viscosity of injected water, which provides more accurate sweep efficiency and oil recovery from reservoirs [7]. The development of smart or responsive polymers that can adapt to changes in conditions, such as temperature and pressure, has been studied. These polymers could find applications in wellbore fluids, seals, and other oilfield components [16]. Within the oil and gas industry, aging pipelines pose various dangers and potential losses due to corrosion-related issues. This has increased research and development efforts to prevent and reduce corrosion. Smart nano-based materials are also employed to create superhydrophobic coatings that protect metallic parts and surfaces from mechanical abrasion, preventing fouling and eventual corrosion. The results indicate that these coatings are highly capable of protecting steel pipes and have garnered significant interest within the industry. With this aim in mind, polymers have been investigated for their ability to protect oil and gas pipelines from corrosion. Anti-corrosive coatings are essential for maintaining infrastructure integrity [17].

Macromolecular additives, including hydroxyethyl cellulose (HEC), guar gum, and xanthan gum, are frequently employed to alter and control the viscosity of fracturing fluids. These polymers can be designed to provide the appropriate rheological qualities under a variety of situations encountered during hydraulic fracturing [18]. The formation must be prevented from encouraging fluid seepage during the operation to ensure effective hydraulic fracturing treatment. Macromolecular additives, which prevent fluid loss, can be mixed into the fracturing fluid formulation to establish a barrier on the fracture faces and minimize fluid loss. Macromolecular compounds used to prevent fluid loss include polymers such as polyacrylamide (PAM) and modified starches [19]. Hydraulic fracturing operations frequently encounter high temperatures and varied salinities in the underground environment. Macromolecular chemistry allows for the production of polymers that maintain their performance and stability in extreme conditions. Crosslinked polymeric systems in which macromolecules are chemically or physically linked to prepare gels also play another vital role in protecting the fracturing fluid from disintegration due to harsh thermal and chemical environments in deep reservoir conditions [20].

**Figure 2:**
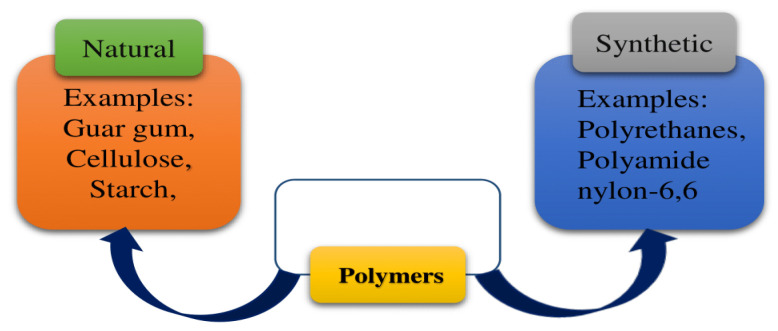
Types of polymers used in fracturing fluids.

Guar gum derived from guar beans is another commonly used natural polymer in fracturing fluids that are prized for managing viscosity and fluid loss. Starch derived from grains is a less expensive alternative to guar gum and cellulose that features comparable rheological qualities and fluid loss control capabilities [21]. Synthetic polymers such as polyurethane and polyamide are manufactured macromolecules produced via chemical synthetic methods. Polyurethane, a flexible synthetic polymer, is used in hydraulic fracturing to prevent abrasion due to its high tensile strength. It is commonly utilized in the production of seal coatings and as a component in downhole tools to improve durability and performance. Polyamide, better known as nylon, is prized for its toughness. It prevents chemical and thermal degradation, thus making it a highly suitable material for various applications within the oil and gas sectors [22]. As shown in Figure 2 below, natural and synthetic polymers play vital roles in developing innovative materials and formulations for hydraulic fracturing operations, resulting in increased efficiency, sustainability, and safety in hydrocarbon extraction from subsurface reservoirs.

Table 1, as described by Berdugo-Clavijo et al., summarizes various types of polymers used in hydraulic fracturing operations categorized by their base material and applications. Guar-based and cellulose-based polymers serve as gelling agents, proppant delivery agents, and fluid loss additives, while acrylamide and acrylic acid-based polymers function as friction reducers and thickeners. In addition, xanthan gum, starch derivatives, polyurethanes, and polyesters find applications in gravel packing, drilling muds, and as foaming agents or thickeners in fracturing fluids [23].

### 2.1. Natural Polymers

These polymers are found in nature and are obtained from plants and animal sources. Typical examples include guar gum, xanthan gum, cellulose starch, etc. Broadly speaking, natural polymers can be classified into two groups: one is those that are derived from plant-based materials (a), and the other is those that are derived from animal sources (b).

#### 2.1.1. Guar Gum (GG)

Guar gum is mainly obtained from the beans of *Cyamopsis tetragonoloba* L. It is a polysaccharide with branching units and contains D-mannopyranosyl sugar units in the backbone connected via β-1, 4-glycosidic linkages. In addition, the backbone is connected to a single-membered D-galactopyranosyl sugar unit via α-1,6-glycosidic linkages [27]. The mannose−galactose ratio (M/G) varies according to geographical origin, ranging from 1.8 to 2:1. Guar gum and polymers derived from guar gum have a wide range of applications, as they are capable of altering rheological characteristics [28,29]. They have numerous uses, including thickening and dispersion., and, in the oil and gas industry, they play a vital role in drilling fluid compositions and preventing corrosion.

##### Physical, Chemical, and Biological Properties of Guar Gum

Like most natural polymers, guar gum can be obtained in molecular weights of large distribution (1–2 Mda). Guar gum solutions exhibit high viscosity at high temperatures due to their hydrodynamic volume and unique intermolecular interactions. Interestingly, guar gum solutions can buffer over a large pH (4.5–10) range and are stable. However, like most other natural polymers of high molecular weight, it is impossible to solubilize guar in organic solvents; only water is capable of solubilizing guar gums. Moreover, due to its poor solubility in organic solvents, guar gum cannot be disintegrated using oils, greases, and other chemicals. Gels and films can also be prepared from guar gum, and guar gums have excellent water-binding capabilities. Numerous hydroxyl groups can create hydrogen bonds. Guar gum’s chemical modification and crosslinking capabilities are known to be excellent [30,31].

##### Polymers Obtained from Guar Gum

Hasan et al. have listed various examples of chemical modification of guar gum involving the replacement of a hydrogen atom in one of the many hydroxyl groups found throughout the macromolecular structures [32,33], as shown in Table 2 [30]. They state that the modification addresses challenges, such as viscosity reduction, altering solubility, reducing turbidity in aqueous solutions, and reducing degradation due to the action of microbes, which limits its long-term application. Chemical modification of guar enables its usage across a wide range of applications. Chemical modifications of guar gum include etherification, esterification, and crosslinking reactions with hydroxyl groups. The most common derivatives of guar gum (see Table 2) include carboxy methyl-guar (CMG), hydroxy propyl guar (HPG), and carboxy methyl hydroxy propyl guar (CMHPG) [31].

**Scheme 1:**
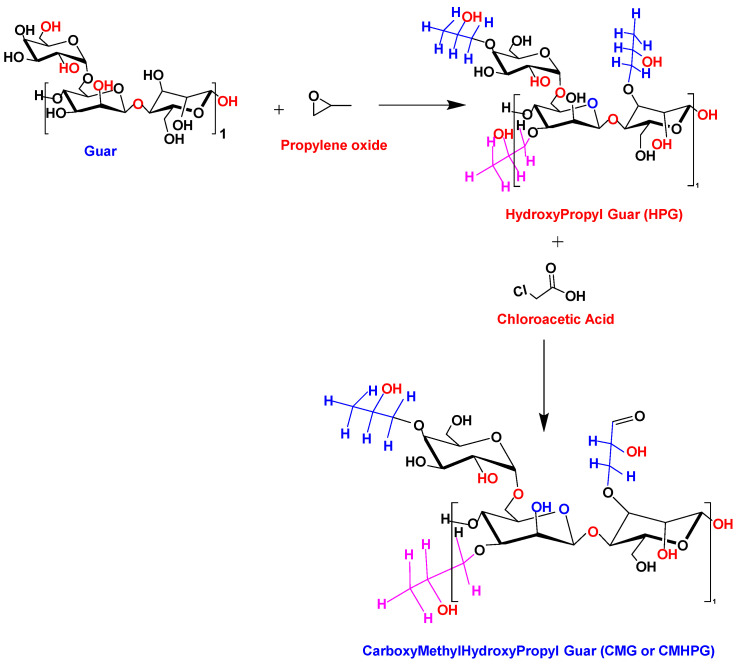
Synthesis of polymers obtained from guar gum [36].

Common polymers, such as HPG and CMHPG, are obtained from guar gum. As they contain carboxylate salts, when the pH of these polymer solutions is reduced to 6–6.5, the polymers have enhanced hydration [34]. The number of insoluble residues in guar gum can vary, and the amount can be altered depending on guar purity and the method used to process it. These insoluble residues limit hydraulic fracturing’s ability to work efficiently. As a result, reducing the number of insoluble residues is important. Towards this goal, guar is often chemically modified to limit the impact of the insoluble residues. Guar can be converted into HPG and carboxymethyl guar (CMG) through alkaline treatment with an oxide, propylene oxide, and chloroacetic acid. Carboxymethyl hydroxypropyl guar (CMHPG) is one of the most efficient guar derivatives [35]. The chemical structures of HPG and CMHPG derived from guar [25,36] are shown in Scheme 1.

##### Employment of Guar Gum and Its Derivatives in the Oil and Gas Industry

The oil and gas industry has utilized hydraulic fracturing technology (HFT) for almost five decades. This technology is commonly employed in the oil and gas industry to enhance hydrocarbon recovery from subterranean formations. Hydrophobically modified guar gum (HMGG) is a novel type of guar derivative created for fracturing fluid compositions [37]. The development has resulted in guar gum-based polymers with hydrophilic and hydrophobic properties. Nonionic polymers work similarly to surfactant micellization by reducing contact between alkyl chains and water molecules, leading to self-association. Hydrophobic polysaccharides, including hydroxypropyl guar, can serve as effective fracturing fluids in EOR operations. When guar and other polysaccharides, such as xanthan gum, are combined, they can produce EOR fluids with superior results in controlled rheology and morphology [38]. In another study, Torres and coworkers found that guar derivatives give rise to excellent surfactant properties, including low surface tension and anti-foaming, even under extreme salinity and temperature circumstances [39]. Hydroxypropyl guar (HPG) is commonly utilized in the oil industry, although carboxymethyl hydroxypropyl guar (CMHPG) is preferred due to its effective hydration and low residue development during fracturing processes [40]. Xiong et al. reported an innovative foam fracturing fluid in which a layered structure without a 3D network was present before the polymer chains were crosslinked. After adding boron, which led to crosslinking, a gel was formed that enhanced the stability and viscoelasticity of the fracturing fluid [41].

The insoluble residues in guar are primarily generated by forming a helix structure due to several unbranched mannose sugar units touching each other and creating a polymannose substructure [42], as reported by Weaver et al. As 6–10% by weight insoluble residue is expected from guar, depending on the method employed to process it, the non-uniform molecular weight distribution gives rise to approximately 6–10% by weight insoluble residue that can cause damage to the proppant pack. Breakers, including enzymes, generate additional residues during preparation, reducing proppant pack conductivity. Precipitate formation can occur over hours or days due to undesirable and premature breaking of the macromolecule’s backbone. Guar derivatives are synthesized by subjecting guar gum in water to elevated temperatures in conjunction with highly alkaline conditions to induce swelling, which enables disruption of the helical structures. This process exposes the polymer backbone to derivatizing agents like propylene oxide, resulting in HPG with approximately 2–4 wt% insoluble residues.

Samanta et al. investigated the effectiveness of surfactants with macromolecules for enhanced oil recovery. They explored the interfacial and rheological properties of guar gum polymer and soapnut shell-derived surfactant designing optimum compositions for flooding experiments [43]. Bahamdan and coworkers prepared grafted poly(oxyalkylene) guar gum and used it in hydraulic fracturing fluid compositions [44]. Olatunde et al. created a water-based drilling fluid that included bentonite, guar gum, polyanionic cellulose PAC, and gum arabic. Guar gum had the most gel strength and stable rheological properties [45]. Table 3 summarizes the various uses of guar gum and its derivatives in the petroleum sector. These polymers are highly effective in corrosion inhibition and enhanced oil recovery (EOR), with concentrations ranging from 0.1 to 1.0 wt%/mol/mmol. They also contribute to viscosity augmentation, which is critical for fracturing fluid performance, with viscosities typically ranging from 500 to 1500 cPs. Modifiers or activators such as borax, calcium chloride, sodium hydroxide, and zinc chloride are often employed to improve the performance of these polymers in various applications in the petroleum industry.

#### 2.1.2. Cellulose and Cellulose Derivatives

**Figure 3:**
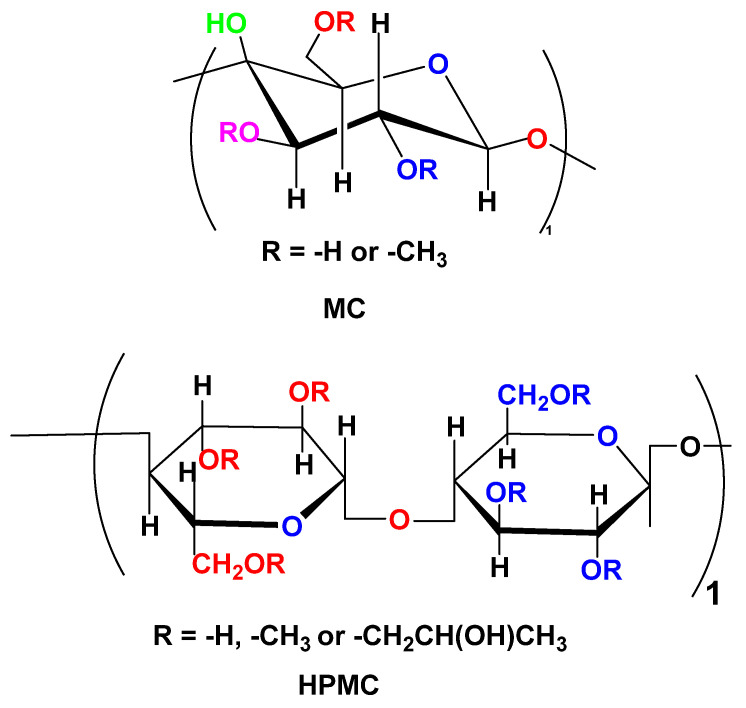
Chemical structure of methylcellulose (MC) and hydroxypropyl methylcellulose (HPMC).

One of the most abundant natural polymers on Earth is cellulose. It occurs widely in the cell walls of marine species and plants, and it is also produced via microbial biosynthesis (algae, fungi, bacteria) [46]. Regardless of the source, cellulose possesses the same chemical structure throughout a polymer chain joined by D-glucopyranosyl units via β-1, 4-glycosidic connections. The hydroxyl groups on C_2_, C_3,_ and C_6_ of glucose units enable the creation of hydrogen bonds with other cellulose molecules [47]. On the other hand, methylcellulose and hydroxypropyl methylcellulose cellulose derivatives with short alkyl substituents show thermogelation behavior and may find applications in the exploration of crude oil [48]. Figure 3 displays the chemical structure of methylcellulose and hydroxypropyl methylcellulose.

**Figure 4:**
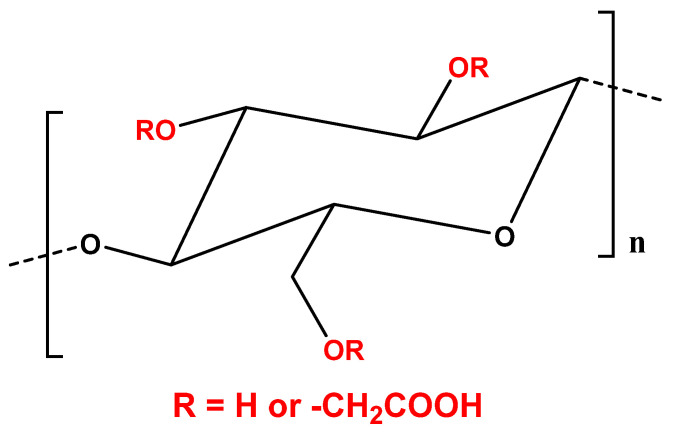
Chemical structure of carboxymethyl cellulose (CMC).

Later, the thermogelation phenomenon was observed in aqueous methylcellulose (MC) solutions where the solution forms a gel at high temperatures and reverts to a liquid state upon cooling [49]. MC’s physical properties and temperature behaviors were thoroughly studied in further research, revealing a lower critical solution temperature of 80 °C [50]. Hydroxypropyl methylcellulose (HPMC), another cellulose derivative, exhibits thermosensitive behavior due to partial methoxyl displacement by hydroxypropyl groups with gelation temperatures determined by the stability between hydrophilic and hydrophobic groups on its molecular chains. Azizov et al. demonstrated that the incorporation of carboxymethyl cellulose within fracturing fluid systems can yield comparable production efficacy and reduced expenses compared to guar gum [51]. The industry is becoming more interested in employing polymers like carboxymethyl cellulose in hydraulic fracturing operations, and thus it is critical to comprehend how cellulose-based polymers biodegrade to create novel enzyme breakers that can effectively combat these kinds of filter cakes. Cellulose is a polymer of repeated glucose units joined by 1,4 bonds. Figure 4 shows that carboxymethyl cellulose is a fiber molecule with randomly placed carboxymethyl groups instead of hydroxyl groups.

One interesting study, as described by Khater et al., involves preparing an in situ hydrogel from enhanced hydroxyethyl cellulose in conjunction with a crosslinker, with the crosslinked product acting as a transient filling gel for longitudinal open hole completion. This cost-effective solution proved to be advantageous for high water-bearing wells, achieving a permeable restoration of 99.9% after gel-breaking by acid [52]. Various oilfields have successfully carried out this technique.

##### Derivatives of Cellulose Utilized in Drilling

Drilling is an absolute necessity in oil extraction, and drilling solutions (sometimes called drilling muds) are an important feature of this process. Drilling muds consist of dense liquid mixtures used in drilling operations and serve various purposes [53]. These tasks involve removing the cuttings, lubricating and cooling the bit used for drilling, controlling the pressure in the formation, and forming a low-permeability mud cake for stabilizing the well wall [54]. While oil-based drilling fluids offer stability in extreme conditions, they pose safety and environmental concerns, leading to a preference for water-based drilling fluids. Rheology, a key property of drilling fluids, directly influences their ability to transport cuttings, and the filtration performance is critical for optimal drilling fluid performance. To improve drilling processes, elasticity regulators and turbidity reductions like starch and cellulose derivatives, polyacrylic acid, lignite resin, and synthetic polymers are used to achieve low fluid loss volumes and optimum cake deposition properties [55,56,57].

##### Use of CMC in Drilling

The most popular drilling fluid component is CMC. Kok et al. investigated the influence of polymer and CMC on the rheological properties and water loss of fluid-based drilling fluids. The ideal concentration of CMC in a fluid directly influences its quality. Fluid loss is gradually reduced when CMC is used. Nevertheless, flocculation occurs when the loading amount is higher than 1 g/350 mL [58]. Jha et al. developed an oil/water emulsion drilling fluid, including CMC and tragacanth gum, as viscosity changes fuel in the dispersed phase and saltwater in the continuous phase. The study found that adding oil and chemicals causes fluid shear-thinning (pseudoplasticity). Drilling fluid’s rheological and filtration qualities remain steady at temperatures up to 70 °C [59].

##### Use of PAC in Drilling

It is common knowledge in the world of drilling that PAC is superior to CMC. One current study examines the application of PAC in oil drilling fluids like salinity drilling fluids and high-density fluids. Yang et al. examined the influence of PAC on drilling fluid viscosity and fluid loss by altering the PAC content, finding a beneficial nonlinear connection between apparent viscosity and PAC concentration. When the PAC content is less than 1.5%, its apparent fluidity rises slowly; however, when the PAC content surpasses 1.5%, the noticeable viscosity increases quickly. In general, low-content PAC can significantly raise the viscosity, enabling the use of low-density drilling fluid for drilling, cleaning, and more efficient drilling operations. According to this study, PAC is a potentially useful addition for drilling liquid that increases viscosity and lowers filtrate volume [60].

##### Use of HEC in Drilling

The impact of HEC’s molecular weight on its performance can be significant. Despite its infrequent application, HEC has been observed to act as a viscosifier in drilling fluids. Ouaer and Gareche [61] investigated the rheological characteristics of HEC solutions at various concentrations and shear rates. The study found that the HEC solution behaved like a Newtonian fluid at low shear rates and thinned at high shear rates. The authors concluded that the concentration of HEC in drilling fluid is semi-solid, based on research findings. The effects of HEC (Mw = 9.5 × 105 g/mol) on the rheological characteristics of a 3% BT solution were investigated by Ouaer and Gareche [62]. Yield stress, apparent viscosity, and elastic modulus all increased when HEC was added to BT suspension, and these effects peaked at a concentration of about 0.1 weight percent. The rheological features of the solution deteriorated when this concentration was reached.

##### Derivatives of Cellulose Used in Fracturing Acidification

There are large oil resources that are challenging to exploit, and the discovery of such deposits will only increase over the next two decades. This petroleum is often found in reservoirs with low permeability, displacement, and a varied composition. Hydraulic fracturing is increasingly being viewed as a viable strategy for improving oil production from these reservoirs [63]. In water-based fracturing fluids, cellulose derivatives are mostly used as gelling agents; however, they can also be used in other fracturing fluids as thickening stabilizers and leakage-reducing agents. Table 4 below describes drilling fluid additives made from cellulose and derivatives such as carboxymethyl cellulose (CMC), which are filtration control agents that minimize fluid loss while maintaining rheology [64]. Polyanionic cellulose (PAC) acts as a viscosifier, increasing fluid viscosity and solid suspension. Hydroxyethyl cellulose (HEC) acts as a rheology modifier to improve fluid performance and hole cleaning. These additives are critical for enhancing oilfield drilling operations, as each contributes unique qualities that improve drilling fluid performance. Table 4 summarizes the properties of various materials used in drilling fluids and their major findings (as described by Liu et al.). Carboxymethyl cellulose (CMC) demonstrates inhibitive properties on clay hydration, with low molecular weight CMC showing slightly lower clay swelling than high molecular weight CMC. Additionally, CMC exhibits a “stickiness” effect, particularly when combined with polyvinylpyrrolidone (PVP), resulting in increased apparent viscosity and shear stress. Polyanionic cellulose (PAC) effectively improves viscosity and reduces filtrate volume; however, when added to bentonite suspension, hydroxyethyl cellulose, in the right amounts, improves the rheological characteristics of drilling fluids.

#### 2.1.3. Starch

**Figure 5:**
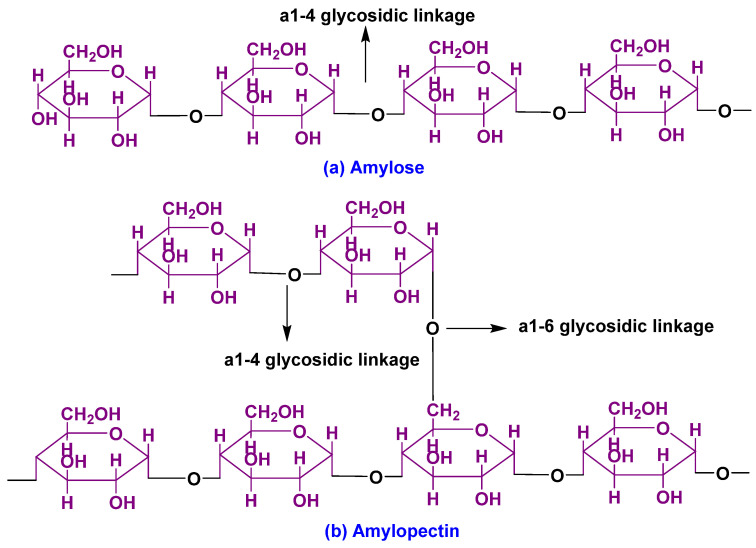
Chemical structure of amylose and amylopectin [74].

The main source of carbohydrates in human meals is starch, a naturally occurring sucrose polymer found in plants [69]. Commercial sources for it include maize, potatoes, rice, wheat, cereals, legumes, and tubers [70]. Starch is a biopolymer formed by chemically linking glucose molecules together via α-1,4-glycosidic bonds [71,72,73]. The biopolymer grains contain varying amounts of amylopectin and amylose, depending on the connections between glucose units. Amylose is a linear polymer composed of long chains of α-d-glucose rings linked via α-1,4-glycosidic linkages, as shown in Figure 5a. Amylopectin is a highly branched polymer developed of α-d-glucose rings with many short chains attached via α-1,6-glycosidic linkages to the macromolecule’s backbone, as shown in Figure 5b.

The percentage of amylopectin and amylose determines the chemical and physical properties of starch. Amylopectin promotes starch adhesiveness, whereas amylose enhances biopolymer gelatinization [75]. Starch is a fundamental material that can be used in many different applications, such as food, glue, coatings, oil drilling fluid compounds, drug transporters, and biofuels [76]. Adding starch to water-flood sites increases cumulative oil production by 6–8% compared to synthetic polymer flooding (commercial polyacrylamide). Improving this biopolymer’s availability, stability, and environmental sustainability would benefit its utilization in EOR applications. Harsh circumstances, including high pressure, temperature, and salt, can reduce the efficiency of native starch in hydrocarbon reservoirs. Abou-alfitooh et al. have summarized various synthetic routes to obtain starch derivatives, including pre-gelatinized, etherified, grafted, and crosslinked starches, which have been used to improve oil extraction processes [77]. Compared to native starch, crosslinked starch made by grafting vinyl monomers onto starch provides excellent rheological characteristics, thermal stability, and microbiological resistance [78] (see Table 5).

El-hoshoudy modified starch by adding a double bond (vinyl type) using the method described by Fang et al. [72,81], which resulted in acryloylated starch. The researcher utilized acryloylated starch to conduct a grafting reaction while employing acrylamide and acrylic acid monomers. The process entailed an emulsion copolymerization reaction with vinyl trimethyl silane. It was observed that the amalgamation of two synthetic polymers enhances the water absorption capability of biopolymers. Employing a linear sandstone model to mimic a reservoir, the enhanced biopolymer composite yielded a 46% recovery factor (S_or_). The improved composite is effective for biopolymer flooding in challenging reservoir settings [71].

El-hoshoudy and Desouky grafted acryloylated starch with acrylamide, vinyl methacrylate, and 1-vinyl-2-pyrrolidone polymers [72]. Polymer flooding trials confirmed the composite’s resilience to high reservoir salinity and temperature-enhancing oil extraction by up to 49% of the remaining saturation favoring biopolymers. Functionalizing starch with thiourea produced an eco-friendly sulfonic acid derivative that was quaternized and polymerized with SiO_2_ NPs through redox initiation. This composite offers a cost-effective environmentally benign solution for the petroleum sector, adhering to sand pore surfaces via electrostatic interactions and boosting oil recovery to 39%. Agi et al. synthesized crystalline starch nanoparticles by employing ascorbic acid, ultrasonic treatment, and nanoprecipitation techniques [79]. The researchers explored the interfacial properties of crystalline starch nanofluid (CSNF) across varying concentrations, NaCl levels, and temperatures. Surface tension decreased with rising nanofluid concentrations, salt contents, and temperatures. Contact angle analysis revealed increased wettability as CSNF concentration rose, transitioning sandstone from hydrophobic to hydrophilic. Core flooding experiments using CSNF and xanthan gum under reservoir conditions resulted in a 23% increase in oil recovery compared to the native biopolymer (XG). Fu et al. synthesized a water-soluble cationic starch via reaction with (2-chloroethyl) trimethyl ammonium chloride (CCC) in NaOH catalysis [80]. The researchers investigated the adsorption of a cationic biopolymer on an oil core matrix, with its pH-dependent impact on sand particle adsorption potentially influencing their hydrophilicity and ionic conductivity to enhance oil recovery. Static adsorption tests revealed that the cationic biopolymer’s saturation adsorption capacity (1.3 mg/g) surpassed that of the HPAM solution (0.35 mg/g). Sand-pack core flood tests demonstrated a significant oil extraction improvement (up to 18% of OOIP) and reduced water cut post-traditional water flooding with modified biopolymer injection. Starch serving as a foundational material has been utilized for creating polymeric additives in the oilfield industry for five decades due to its unique structure and reactivity facilitating the development of various oilfield chemicals. Numerous modified starch derivatives have been synthesized and commercially deployed for oilfield applications [82,83,84].

In particular, these applications mainly contain pregelatinized starches, which are employed in oilfield treatments in China due to their cost-effectiveness [82,83,84]. The process of obtaining pregelatinized starch involves gelatinizing native starches typically in acidic or alkaline environments. High temperatures are essential for breaking hydrogen bonds in starch granules, with the gelatinization temperature varying by starch source. Using sulfuric or sodium hydroxide hydrolysis, Zhang prepared pregelatinized starches for drilling mud [84]. Corn starch was treated with precise concentrations of aqueous sulfuric acid or sodium hydroxide for a set time. The resulting starch paste underwent hydrolysis by H_2_SO_4_ or NaOH, followed by neutralization, alcohol precipitation, filtration, purification, vacuum-drying, and powdering, yielding pregelatinized starch. Zhang proposed its use as a fluid-loss control agent in saline drilling fluid systems [85]. Pregelatinized starches show promise as non-damaging filtrate reducers in water-based fracturing fluids, addressing concerns regarding the formation damage of conventional non-degradable fluid-loss reducers. Unlike silica flour and clay, pregelatinized starches are fully degradable and leave no residue. Williamson and Allenson’s research on pregelatinized potato and corn starch demonstrated their effectiveness in reducing spurt loss and controlling fracturing fluid leak-off, with similar studies lacking in China [86].

### 2.2. Synthetic Polymers/Man-Made Polymers

**Figure 6:**
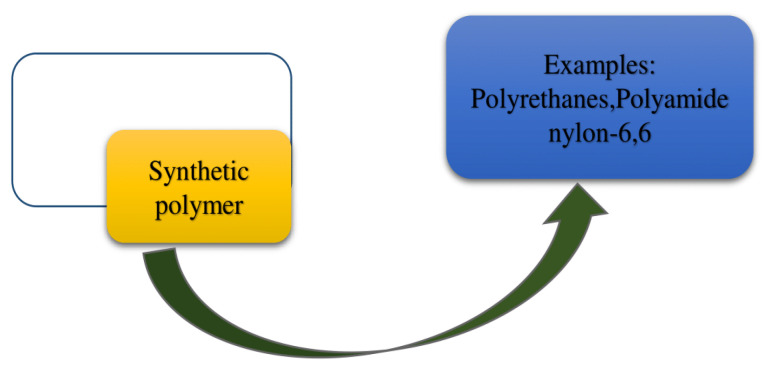
Types of synthetic polymers.

Macromolecules that are manufactured in the laboratory by polymerization of smaller or simple chemical molecules (monomers) are called synthetic polymers. Various synthetic polymers are shown below in Figure 6, such as polyurethanes, nylon, etc.

The comparison of natural polymers over synthetic polymers is listed below in Table 6.

#### 2.2.1. Polyurethanes (PUs)

Polyurethanes (PUs) are a class of polymers that are synthesized via the reaction of organic isocyanates and compounds featuring hydroxyl groups. The versatility of polyurethanes allows for precise design based on the selection of the initial monomer. This customization allows the creation of polyurethanes with specific characteristics, including thermosetting or thermoplastic behaviors such as rigidity or flexibility and solidity or foaminess [89]. Rigid polyurethanes are utilized for insulation due to their beneficial attributes, which include low thermal conductivity and high electrical resistance. In offshore applications, helical polyurethane sheaths are affixed to pipelines to mitigate vortex-induced vibration, supporting horizontal current forces’ dissipation [90]. Aromatic polyurethanes display reduced light stability, shifting from off-white to reddish-brown upon exposure. They are vulnerable to chloride attack, especially in solutions with hydrochloric acid (HCl) and hypochlorous acid (HOCl), forming amine salts and polyols [89]. For use in the manufacture of high-performance coatings [91], elastomers [92], and carbon fiber composites [93], Pus have been modified into functionalized or crosslinked varieties. High-performance Pus, including fluoropolymers and epoxies, serve as coatings in the oil and gas industry for various applications like ship hulls and offshore equipment [94]. Xiang et al. utilized a hexadecyl-functionalized low-defect PU. Graphene nanoribbon-incorporation enhanced gas barrier properties, reducing PUs nitrogen gas diffusivity by three orders of magnitude with 0.5 wt% loading [95]. Yao et al. presented a novel piezoresistive sensor with ultrahigh pressure sensitivity using a shattered Graphene-nanosheet-coated PU sponge microstructure design [96]. Polyurethanes are commonly formed by isocyanates reacting with polyols catalytically or photochemically [97]. The key aspects of preparing polyurethanes, as is the case for a large number of polymers, largely depend on the source of monomers. Table 7 highlights the pros and cons of utilizing alcohol-bearing monomeric units from certain sources.

**Figure 7:**
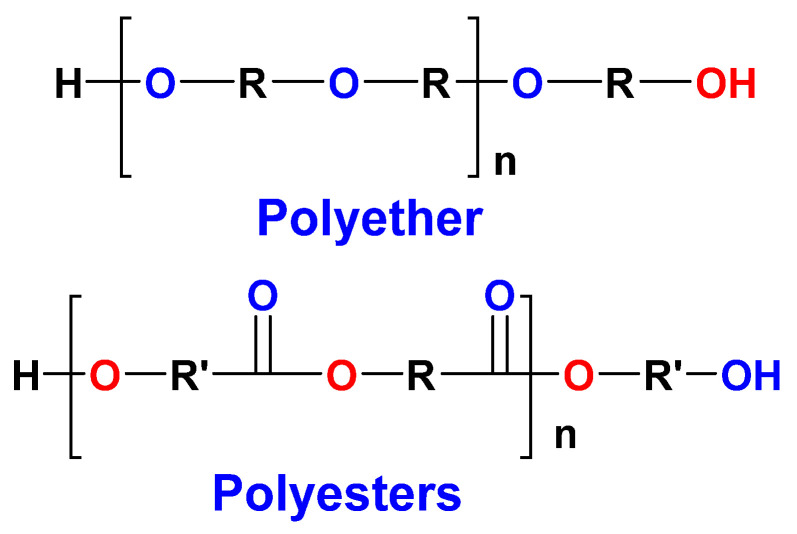
Basic polyol structures.

Isocyanate and polyol compounds form polyurethanes, each with at least two isocyanate and hydroxyl groups. Properties can vary with different polyols and isocyanates, enabling versatile applications due to their crosslinked three-dimensional network structure [98,99]. Polyols utilized in polyurethane synthesis typically feature multiple hydroxyl (–OH) groups. Polyether polyols result from copolymerizing propylene oxide and ethylene oxide. Polyester polyols resemble polyester polymers synthesized analogously. Additionally, specific polyether polyols like poly (tetra methylene ether) glycols can be synthesized through tetrahydrofuran polymerization, serving in highly efficient elastomeric applications [100,101]. Rajendran et al. reported the synthesis of isocyanate-terminated prepolymers using polytetrahydrofuran, providing detailed characterization studies [102]. Polyols are commonly employed as mixtures with similar but varying molecular weights and –OH group numbers. Industrial-grade polyols are precisely measured to ensure consistent properties crucial for tailored polyurethane characteristics, such as rigidity or flexibility [103]. Basic polyol structures are shown below in Figure 7.

**Scheme 2:**
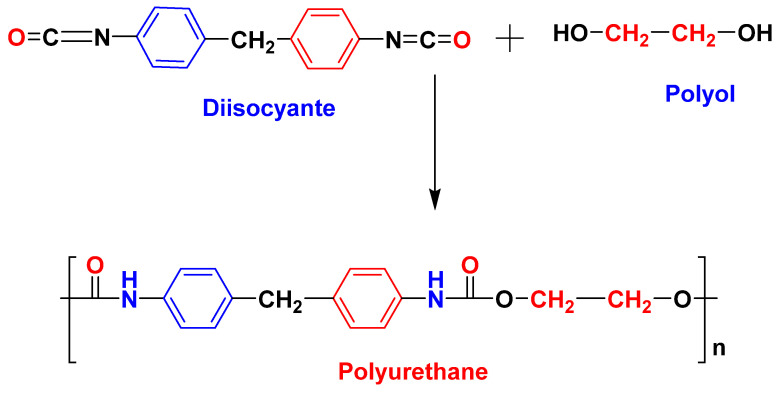
Synthesis of Polyurethanes [89].

A variety of routes can create PUs. The most effective approach involves reacting a polyol (an alcohol with several hydroxyl groups) with a diisocyanate [98]. Scheme 2 shows the production of a distinctive polyurethane. Different additives and catalysts may be utilized to produce PUs.

**Scheme 3:**
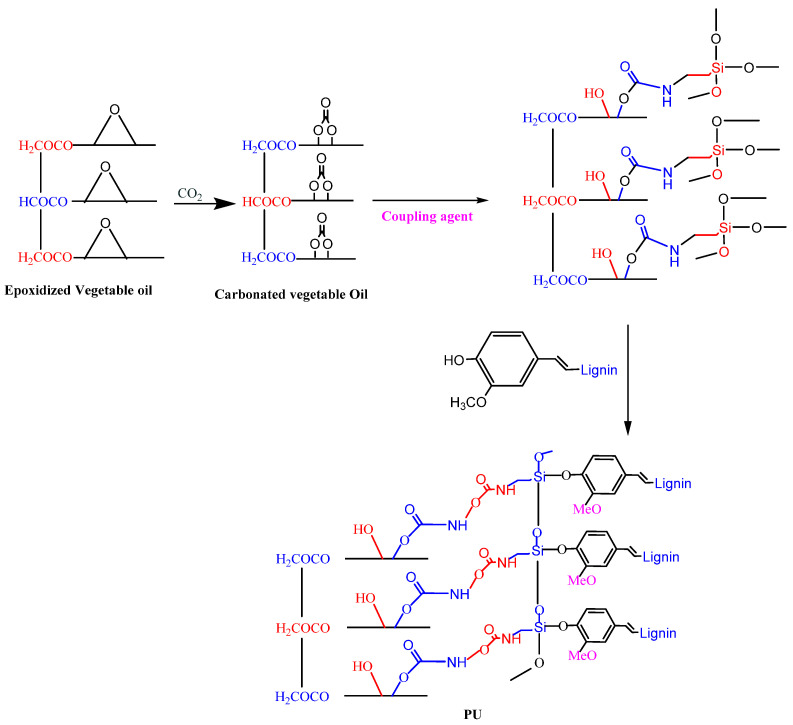
Synthetic approach for PU preparation employing castor oil and lignin in an isocyanate-free process [89].

A study synthesized sustainable polyurethane from carbonated soybean oil, 3-aminopropyltriethoxysilane, and lignin via a non-isocyanate route [104]. One method used oligomeric polybutadiene diisocyanate for lignin-based PU synthesis. Another employed lignin-aminated polyol and diphenyl diisocyanates [105,106,107]. Reports suggest that non-isocyanate-based PU properties depend on lignin content, which affects crosslinking and material modulus. A synthetic route to prepare sustainable polyurethanes is highlighted below in Scheme 3, which has been described by Akindoyo et al. [89].

#### 2.2.2. Polyamides

**Scheme 4:**
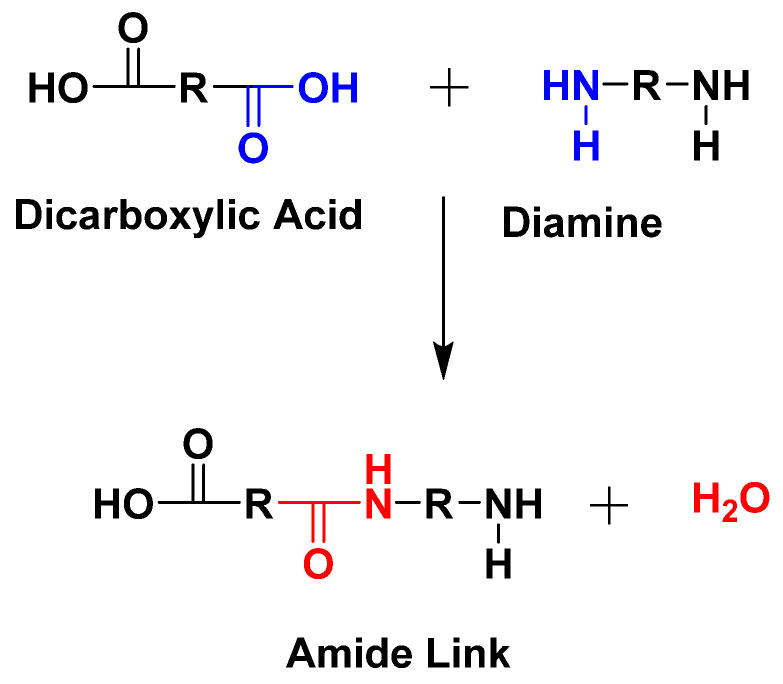
Condensation reaction between a dicarboxylic acid and diamine to form a polyamide.

Polyamides, known as nylons, contain amide groups in their main chain, as seen in nylons 6 and 6, 6. They form through the reaction of diamine molecules with dicarboxylic acid molecules, resulting in amide linkages by condensation with water as a byproduct, as depicted in Scheme 4.

In investigating post-crack creep in synthetic fiber-reinforced concrete under flexure [108], we compared the performance of polypropylene fibrillated fibers and polyamide single filament (nylon 6), finding that both materials exhibited a limited post-crack strength, with polypropylene sustaining only 24.9% and polyamide 38.3% of average residual strength, indicating faster-but-shorter duration creep in polyamide-reinforced concrete compared to polypropylene. Table 8 addresses the use of synthetic polymers such as polyurethane and polyamide in drilling fluids. These polymers have high corrosion inhibition efficiency (ranging from 90% to 85%) and Enhanced Oil Recovery (EOR) capabilities (ranging from 60% to 55%). Concentrations vary from 0.5 wt% to 1.5 wt%. Viscosity ranges from 500 to 1500 cPs depending on polymer and concentration.

### 2.3. Hyperbranched Macromolecules

While working on a new route to manufacture polyphenylene macromolecules for rheological uses, Kim and Webster made an interesting discovery at DuPont in the early 1990s. They were attempting to polymerize 3,5-dibromobenzene boronic acid via Pd-catalyzed condensation to form polyphenylene dendrimers in a one-pot fashion. However, they discovered that the resultant polyphenylene had a considerable number of linear units and the usual branched units in its bonding architecture; it was polydisperse rather than monodisperse, as they had expected. Once they realized they had created macromolecules with new physical architectures, the resultant polyphenylene products were labeled as hyperbranched polymers [109]. Subsequently, this area of synthetic polymer chemistry gradually gained pace, and over the past three decades, there have been numerous studies carried out that have involved the synthesis of hyperbranched macromolecules of various chemical functionalities, such as polyaromatics, polyamides, polyesters, polyamidoamines, and polyethers in bulk, to name a few.

**Scheme 5:**
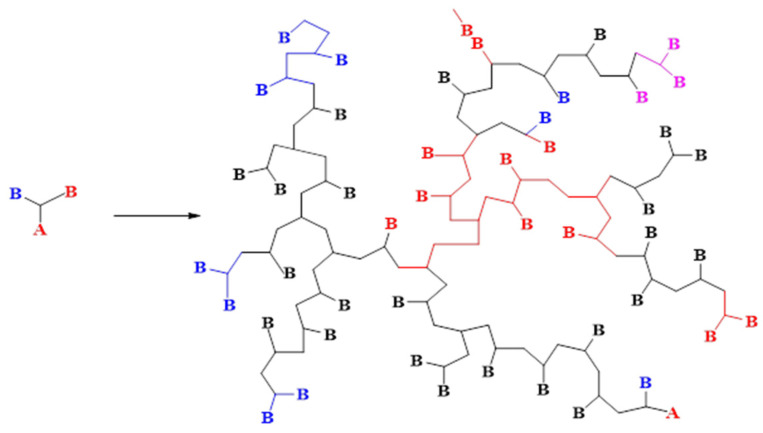
Preparation of a hyperbranched polymer via single monomer methodology (SMM) route using an AB_2_ monomer.

Traditionally, the various synthetic routes employed to produce hyperbranched polymers can broadly be covered by two established methodologies. The first is Single Monomer Methodology (SMM), which primarily uses an AB_2_ monomer, as shown in Scheme 5 below. By far, this is the most popular method of producing hyperbranched polymers, as can be seen from studying the chemical literature. Shen et al. conducted an investigation that reacted 5-aminoisophathalic acid as an AB_2_ monomer with trimesic acid as a core molecule while using the condensing agent diphenyl 223-dihydro-2-dihydro-2-thioxo-3-benzoxazoyl phosphate (DBOP) [110]. When designing the monomers for this kind of synthesis, it is vital that functionality A only reacts with functionality B, with both B functionalities needing to be of equal reactivity to ensure the successful formation of the hyperbranched polymer.

**Scheme 6:**
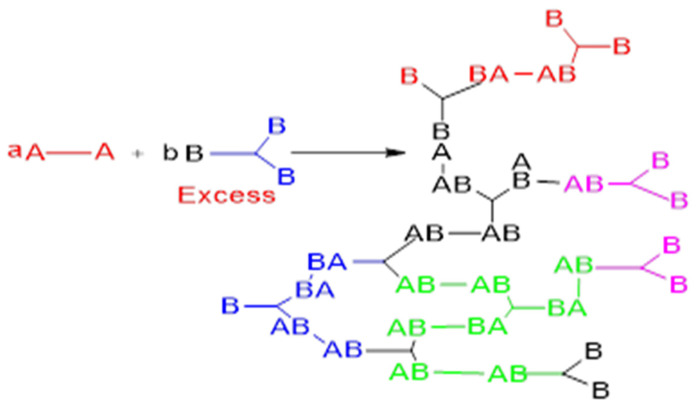
Preparation of a hyperbranched polymer via double monomer methodology (DMM) route using an A_2_ and B_3_ monomer combination.

The other methodology that can be employed to produce hyperbranched polymers is Double Monomer Methodology (DMM), as demonstrated in Scheme 6 below.

Hyperbranched polymers exhibit intricate three-dimensional structures and modified terminal functional groups. They amalgamate dendritic macromolecules with a multi-terminal design and a high molecular weight linear polymer. Altering the active end groups can modify their physical and chemical traits. Their reduced viscosity stems from a highly branched structure that enhances solubility, rheology, and resistance to temperature and salt compared to linear polymers. Despite being introduced three decades ago, studies on hyperbranched polymers were limited initially. However, their industrial applications, including biomedical, anti-fouling, and anti-corrosion materials, have increased over time [111,112,113,114,115].

#### 2.3.1. Synthesis of Hyperbranched Macromolecules

Hyperbranched polymers can be produced using diverse monomers and evolving techniques. However, the most popular methods employed in recent years are condensation, ring-opening, and free radical polymerization.

##### Condensation Polymerization

**Scheme 7:**
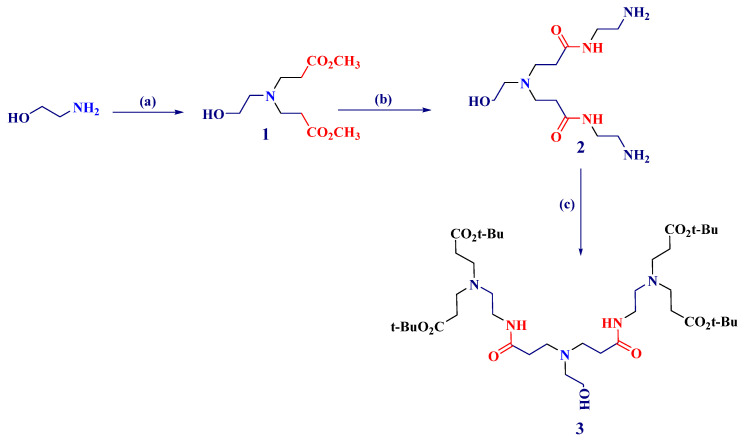
AB_2_-type monomers undergo condensation polymerization to form hyperbranched polymers [117].

Condensation polymerization forms polymers from multifunctional monomers via repeated condensation reactions releasing water and small molecules. This reversible and progressive process steadily increases molecular weight. ABn-type polycondensation initially employing AB_2_-type monomers is common for hyperbranched polymer synthesis, promoting increased monomer utilization. Monomers commonly employed include propylene glycol, trimethylolpropane, and AB_3_-type monomers [116], as well as AB_4_-type monomers such as ethylenediamine, pentaerythritol, and p-diaminobenzene. Rousseau et al. synthesized a hyperbranched polymer from ethanolamine and methyl acrylate [117]. The reaction process is depicted in Scheme 7.

Hyperbranched polymers offer good yields and involve moderate reaction conditions. In this particular synthesis protocol, the reaction involves multiple steps that require purification of the products before proceeding to the next phase. Synthesis is expensive, making it unsuitable for industrial uses. The reaction yields ester groups prone to hydrolysis at elevated temperatures, limiting suitability. Condensation polymerization is a process with two main benefits: high efficiency and a broad range of monomer sources. However, because the products hydrolyze readily, applicability is restricted.

##### Ring-Opening Polymerization

**Scheme 8:**
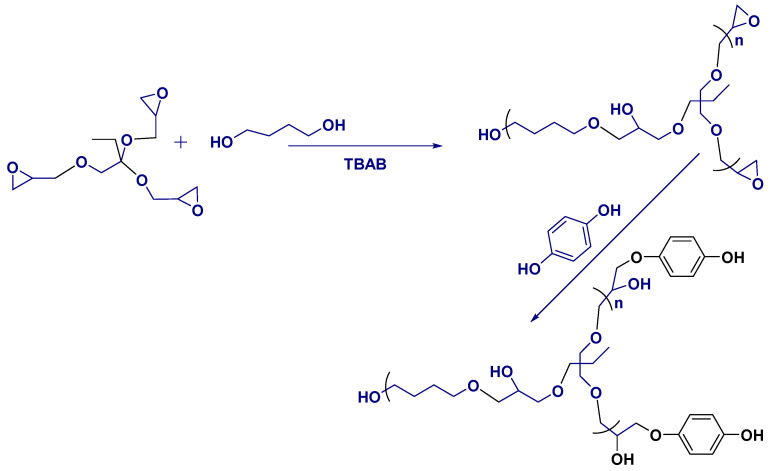
Synthesis route for hydroxyl-containing hyperbranched polymer via dual monomer approach [118].

The process by which a ring-opening addition transforms cyclic compound monomers into linear polymers is known as ring-opening polymerization. Tetrahydrofuran, caprolactone, ethylene oxide, and cyclic carbamate are heterocyclic chemicals employed as monomers in the ring-opening polymerization process to create hyperbranched polymers. Polyester, polyether, and hyperbranched polyamide have all been created using ring-opening polymerization. Xing et al. utilized successive ring-opening polymerization and condensation reactions, employing tetrabutylammonium bromide as a phase transfer catalyst to synthesize a hydroxyl-containing hyperbranched polymer from specific monomers [118] via employment of double monomer methodology. Scheme 8 depicts the process of the reaction.

The reaction provides major advantages, as evidenced by examining the two hyperbranched polymers’ synthesis pathways. Hyperbranched polymer synthesis from epoxy monomer requires a one-step reaction, offering high molecular weight and milder conditions than polycondensation; it also produces fewer byproducts. A non-negligible drawback of this reaction is that it requires an epoxy-based monomer as one of its raw components, limiting reactive monomers’ availability.

##### Free Radical Polymerization

**Scheme 9:**
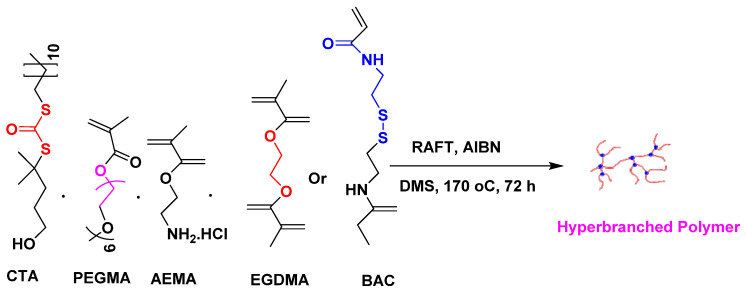
Radical polymerization for hyperbranched polymer synthesis [119].

Monomers for free radical polymerization typically comprise alkenes with unsaturated double bonds. In this reaction, the double bond undergoes opening, initiating addition reactions between molecules and leading to macromolecule formation. Marasini et al. employed N, N-dimethylformamide solvent in reversible addition−fragmentation chain transfer polymerization using 2,2-azobis(2-methylpropionitrile) as the initiator and 4-cyano-4((dodecylsulfanylthiocarbonyl)sulfanyl)pentanol, polyethylene glycol monomethyl ether methacrylate, 2-aminoethyl methacrylate hydrochloride, and ethylene glycol dimethacrylate or N, N-bis(acryloyl)cystamine as monomers [119]. Scheme 9 illustrates the reaction process.

Radical polymerization enables diverse monomer usage, facilitating higher molecular weight hyperbranched polymer synthesis via vinyl-active group interactions. This process produces hyperbranched polymers, which require numerous vinyl groups in the monomer in contrast to linear polymers. Unfortunately, this approach frequently results in gel formation during polymerization, which lowers the rate at which monomer is converted during polymerization. Furthermore, ^1^H NMR spectroscopy is incapable of measuring the degree of branching in polymers. Creating polymers with fixed molecular weights is challenging due to high molecular weight dispersion. Recently, a review article that can be described as concise but comprehensive by Zhang and Yu extensively covered the various synthetic methods to prepare hyperbranched polymers (particularly as described in Table 9), compiling the main positive and negative features of the various methods available to produce hyperbranched polymers [120].

#### 2.3.2. Use of Hyperbranched Polymers in the Oil and Gas Industry

Polymers serve as oil displacement agents, improving recovery by enhancing fluid viscosity, oil−water ratio, and displacement fluid sweep volume. This is an area within the oil and gas industry where selection and use of macromolecules in a judicious manner could lead to enhancement of results. The employment of hyperbranched macromolecules in the oil and gas industry is still in its infancy, but we are beginning to witness the emergence of employing hyperbranched polymers within the oil and gas industry in various areas, as can be seen in an interesting review article by Pan et al. that highlights research and development efforts in this area [121]. Lai et al. synthesized a temperature and salt-resistant hyperbranched polymer from various monomers, achieving a 23.51% increase in oil recovery at 80 °C [122]. Chen et al. synthesized HPG-HPAM-C10AM, a hyperbranched polymer with a spherical parent nucleus and zwitterionic functional chain segments. The spherical parent nucleus, HPG, was formed via a ring-opening reaction of 1,1,1-trihydroxymethylpropane with glycidyl. HPG-HPAM-C10AM exhibited higher viscosity retention (70%) than linear polymer HPAM (40%), suggesting superior shear resistance [123]. Zhang et al. synthesized H-PAMAM, a hyperbranched polyamidoamine with good demulsification properties, utilizing methyl acrylate and ethylenediamine in a one-pot multi-step technique. Within 30 min, the demulsification equilibrium was established and 91% of the oil was removed [124]. This approach eliminated the purification step, thus reducing time and cost.

The corrosion inhibition efficiency and improved oil recovery (EOR) capabilities of various hyperbranched polymers synthesized using condensation, radical, and ring-opening polymerization techniques are shown in Table 10 below. Polymer A, produced via a condensation method, has an 85% corrosion inhibition effectiveness and 60% EOR at a suggested concentration of 1–5 wt%. Polymer B, prepared using radical polymerization, has a greater corrosion inhibition efficiency of 90% and EOR of 70%, with concentrations ranging from 2 to 10 mol%, whilst Polymer C, produced via ring-opening polymerization, exhibits a corrosion inhibition efficiency of 75% and an EOR of 50% at concentrations ranging from 0.5–2 mmol/mol. Table 10 highlights a growing number of applications that are beginning to utilize hyperbranched polymers within the oil and gas industries.

## 3. Chemical Structure and Properties of Linear Polymers Used in the Oil and Gas Industry

**Figure 8:**
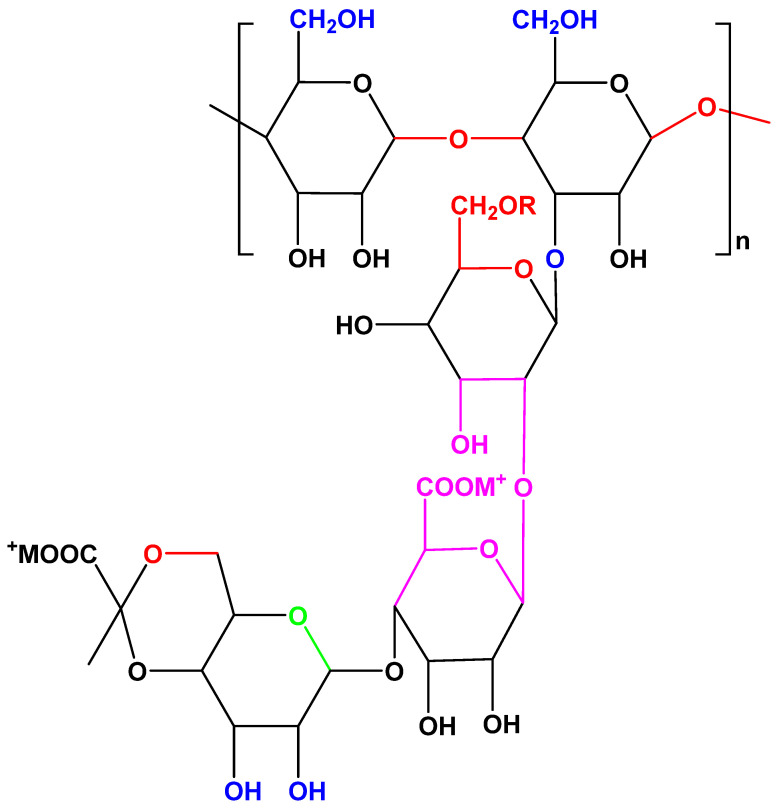
Chemical structure of xanthan gum [8].

Almost all natural polymers utilized in the oil and gas industry are generally based on a physical architecture that can be best described as linear with small amounts of branching; however, the degree of branching in natural branched polymers cannot be described as hyperbranched as the degree of branching is only a few percent and the physical architecture is mostly heterogeneous rather than being homogenous, as is the case with man-made dendrimers and, to some extent, with man-made hyperbranched polymers. In addition, most of the popular man-made polymers, such as PAM and HPAM, are also linear in physical architecture. The main factor governing natural polymers’ properties is their chemical architecture. For example, xanthan gum consists of glucose, mannose, and glucuronic acid units forming an anionic heteropolysaccharide with a linear β-(1–4)-d-glucopyranose glucan backbone. Each glucose at the C-3 position connects to a trisaccharide side chain, as shown below in Figure 8. Pyruvic acid and acetyl groups confer negative charges to xanthan gum [127]. Xanthan gum has a molecular weight (MW) of an approximate range of 2 × 10^6^–2 × 10^7^ Da and exhibits a shear-thinning effect due to its stiffness and intermolecular interactions. Furthermore, the polymer’s side chains contain acetate and pyruvate groups, xanthan gum’s high molecular weight enables thickening, while stiff polymer chains resist shear, salinity, and divalent ions.

In aqueous solutions, xanthan gum adopts an order-to-disorder conformation, transitioning to a rigid state in the presence of ions.

### 3.1. Physiochemical Properties

**Figure 9:**
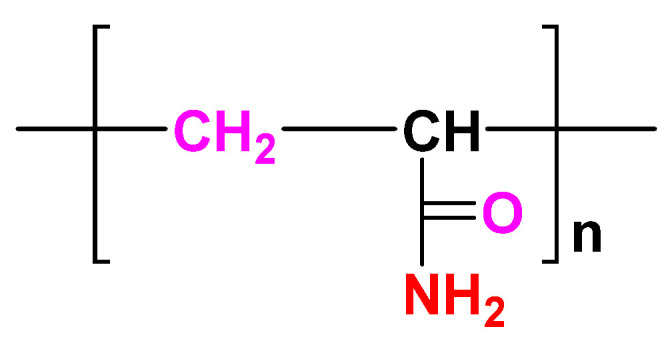
Molecular structure of polyacrylamide (PAM) [128].

Xanthan gum powder is a free-flowing white-to-cream-colored substance that is soluble in hot and cold water but not in most organic solvents. Its high viscosity makes it an effective stabilizer and thickener. Polyacrylamide (PAM) is favored for EOR due to its high molecular weight (>1 × 10^6^ g/mol) and nonionic nature when unhydrolyzed (see Figure 9).

**Figure 10:**
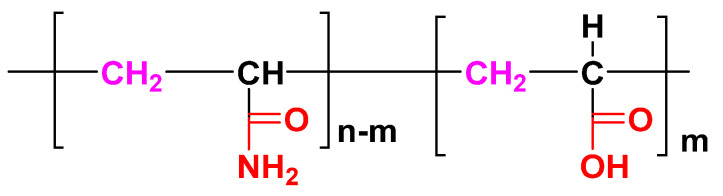
Molecular structure of hydrolyzed polyacrylamide (HPAM) [128].

PAM is widely utilized in gel formation including subsurface and surface crosslinked polymer gels due to its affordability and abundant carboxyl and amide groups [129]. HPAM is frequently employed in field polymer floods due to its ability to withstand mechanical forces, bacterial resistance, and cost-effectiveness. The polymer depicted in Figure 10 is synthesized by copolymerizing sodium acrylate with acrylamide or partially hydrolyzing polyacrylamide and polyacrylic acid [128].

**Scheme 10:**
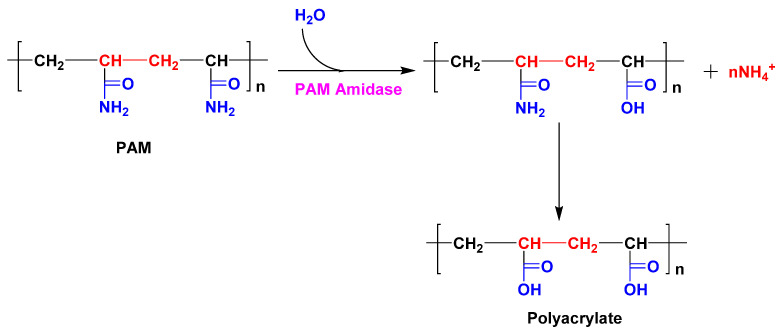
Microbial utilization of PAM and HPAM via enzymatic amidase hydrolysis [23].

Elevated temperatures or alkaline conditions induce hydrolysis, yielding stable partially hydrolyzed polyacrylamide (HPAM). Carboxyl and amide groups enable crosslinking, forming gels with robust mechanical properties. Recently, the utilization of PAM and HPAM in hydraulic fracturing fluids surged, especially in North America [130]. As a result, it is critical to understand the biodegradation by enzymes for breaking down polymer filter cakes prepared from PAM and HPAM. PAM is a polymer with a high molecular weight that is synthesized from acrylamide; it degrades via a certain pathway, as shown in Scheme 10, which has been described in detail in an extensive review by Berdugo-Clavijo et al. PAM’s high molecular weight and stable carbon backbone make it resistant to microbial degradation [131,132]. Microbial utilization of PAM and HPAM has been noted since the late 1990s. PAM and HPAM’s amide (-NH_2_) side groups can be degraded by microbes and transformed into ammonium (NH_4_^+^)**,** providing a nitrogen source for microbial growth. PAM and HPAM are used as nitrogen sources by both aerobic and anaerobic microbes [23].

Amide side group hydrolysis of PAM or HPAM is enzymatically catalyzed by amidase [133,134] (Scheme 10). Chemical analyses indicate microbial conversion of PAM amide to carboxylic acid [135,136], resulting in polyacrylate macromolecules. Deamination of PAM/HPAM’s -NH_2_ groups maintains viscosity and molecular weight [131]. Therefore, amidases are not suitable enzyme breakers for PAM/HPAM.

**Scheme 11:**
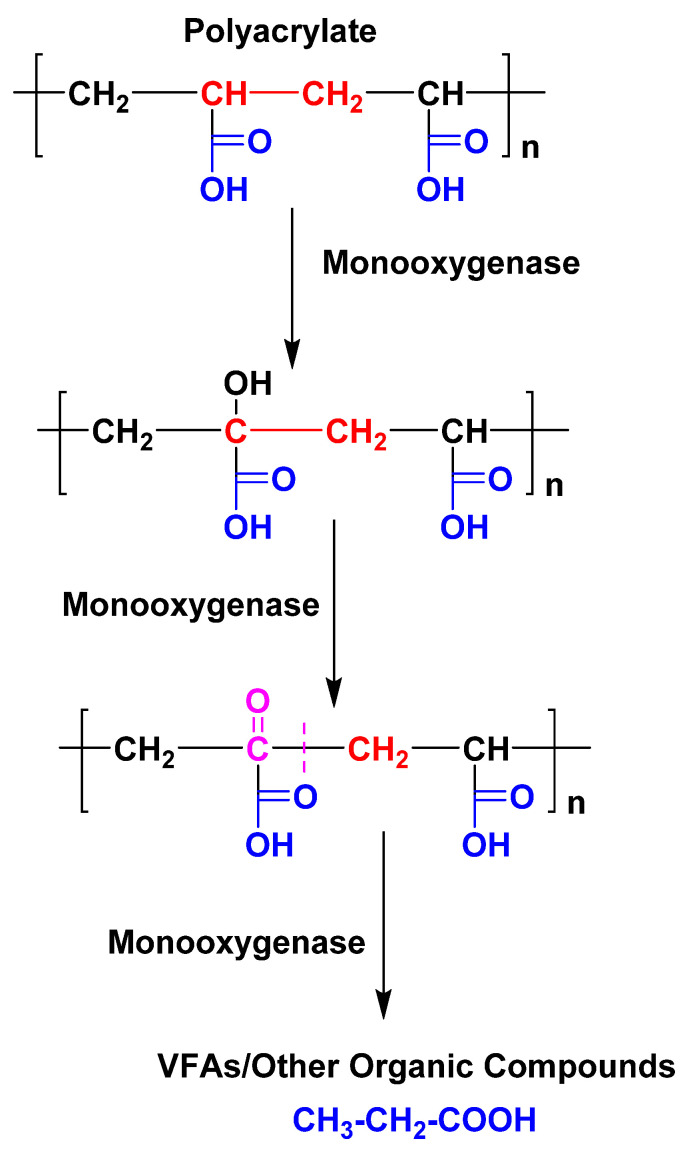
The proposed biodegradation of PAM involves oxidation catalyzed by monooxygenase enzymes [142].

Microbial utilization of PAM or HPAM as a carbon source is complex. Some studies show partial biodegradation, but the lack of controls makes assessment challenging. Nakamiya and Kinoshita found 20% PAM degradation in 27 h [137]. Wen et al. observed that two Bacillus isolates degraded 70% of PAM in 96 h, as assessed via a starch-cadmium iodine assay measuring amide group removal [138]. Bao et al. observed a 14% removal of HPAM by bacterial cultures from oilfield wastewater. Microbial communities in a mixed aerobic and anaerobic reactor reduced HPAM viscosity by 78% [139]. Two molecules with lower molecular weight than HPAM were identified. Other recent investigations [140] found the existence of volatile fatty acids, such as propionate, acetate, and formate, in anaerobic microbial cultures using HPAM. These fatty acids are believed to accumulate due to HPAM biodegradation. Sang et al. investigated laccase and dehydrogenase activity in bioreactors modified with glucose, urea, and conditioned with HPAM [141]. In this study, laccase activity remained constant regarding HPAM concentration, while dehydrogenase activity inversely correlated with HPAM concentration. Oxygenase enzymes facilitate HPAM biodegradation by adding functional groups (Scheme 11).

**Scheme 12:**
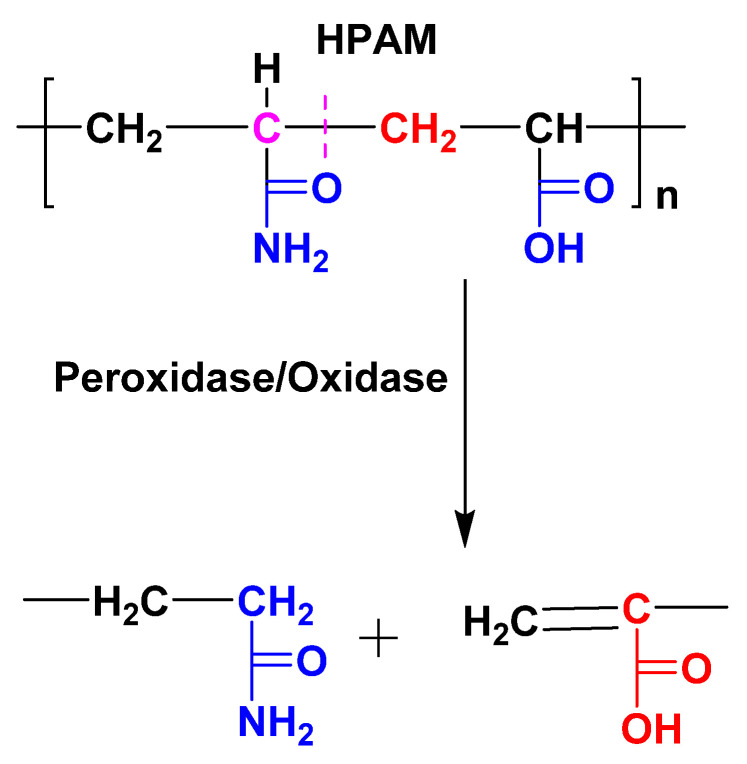
Mechanism of PAM degradation by radical-forming enzymes [142].

Additional investigation is necessary to confirm the existence of enzymes in PAM-degrading cultures and their potential application in oilfield systems. A recent study [142] raises concerns about the use of HPAM or PAM polymers as a carbon source. Inoculating microbial communities from activated sludge and oilfield water under thermophilic conditions did not decrease polymer viscosity with PAM or HPAM as sole carbon sources. Enzymes are directly added to solutions that degrade PAM or HPAM. To destroy PAM, Gupta et al. invented an enzyme breaker known as asparaginase. The enzyme deaminates PAM’s amide group, breaking the polymer and reducing viscosity [143]. Extracellular oxidases and peroxidases can break down PAM/HPAM by generating free radicals that cleave the polymer’s carbon backbone (see Scheme 12).

Ramsden et al. observed xanthine oxidase degradation of 0.5% PAM at 20 °C [144]. Nakamiya et al. demonstrated Azotobacter beijerinckii HM121’s hydroquinone peroxidase degrading PAM into lower molecular weight polymers with tetramethyl hydroquinone and hydrogen peroxide at 30 °C [145]. Hydroquinone peroxidase generates hydroxyl radicals which react with tetramethyl hydroquinone, disrupting PAM’s carbon chain via hydrogen abstraction. Gilbert et al. observed horseradish peroxidase (HRP) catalyzing HPAM degradation at 37 °C using hydrogen peroxide, resulting in significant viscosity and molecular weight reduction [146]. Studies demonstrate that oxidases and peroxidases forming free radicals can degrade PAM or HPAM in oil reservoirs at mesophilic temperatures (20–37 °C). Further research is needed to assess enzyme compatibility with reservoir chemicals.

### 3.2. Macromolecules Impact on Physical Properties

#### 3.2.1. Viscosity

**Figure 11:**
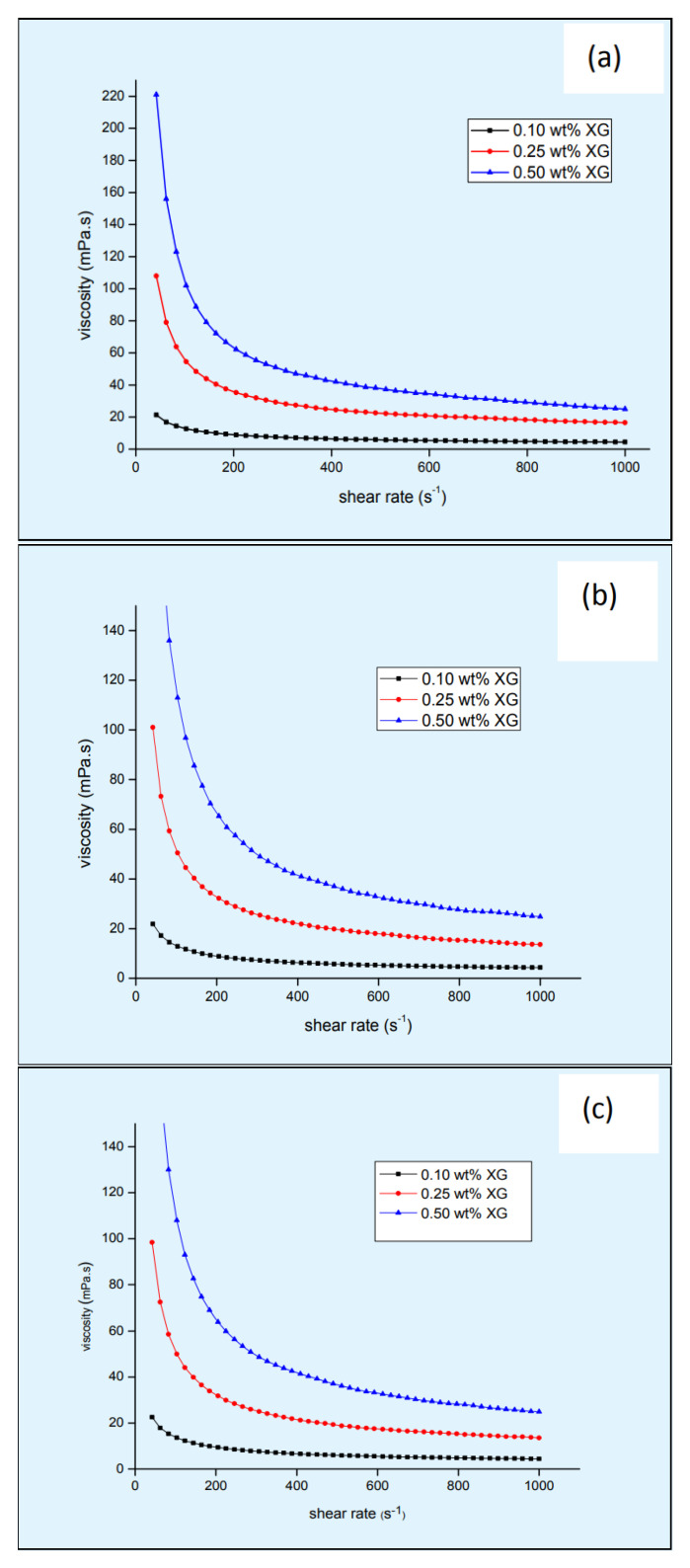
A graph depicting the relationship between viscosity and shear rate for various concentrations of xanthan gum at three different temperatures: (**a**) 303 K, (**b**) 313 K, and (**c**) 323 K [147].

The viscosity of polymers forming gels in fracturing fluids like xanthan gum is a key characteristic. The flow curves for xanthan gum at various shear rates and temperatures are illustrated in Figure 11. Viscosity decreases with increasing shear rates, indicating shear thinning in Xanthan gum solutions across concentrations (0.10 wt%, 0.25 wt%, 0.50 wt%) and temperatures (303 K, 313 K, 323 K), as depicted in Figure 11. Polysaccharides in water form ordered structures that feature large molecules clustering and entangling, leading to high viscosity at low shear rates. Higher xanthan gum concentrations increase viscosity through intermolecular interactions and entanglements, forming larger macromolecules [147].

Polymer chains uncoil and partially align at high shear rates, resulting in lower constant shear viscosity. Xanthan gum solutions are very pseudoplastic. When a shear rate is applied, they exhibit shear-thinning behavior but then quickly return to their initial viscosity. Mansha et al. synthesized a novel procaine-based Gemini zwitterion incorporated PVDF [148]. Zhong et al. synthesized a hydrophobic terpolymer with NaAMPS and VN. They evaluated its stability in sodium chloride brine at 80 °C for 90 days. The HMAP retained 88.3% of its initial viscosity. Incorporating VN chains in terpolymer increases chain stiffness and prevents amide group hydrolysis at high temperatures, resulting in a small amount of polymer breakdown [149]. Mansha et al. studied the viscosification of amidosulfobutaine and zwitterionic gemini surfactants [150]. Maia et al. studied the rheological behavior of polyacrylamide and acrylamide copolymer with N-N-dihexylacrylamide in a porous media. They evaluated the effect of salt on these systems and their prospective application in EOR from core flooding studies. The addition of NaCl increased the viscosity of HMAP, and the F_R_ of the copolymer solution was higher than that of commercial PHPA [151]; this suggests that the copolymer could improve sweep efficiency. Li et al. examined the rheological properties of PHPA, HMAP, and cyclodextrin-modified HMAP-CD solutions in sandstone porous media. Results showed that HMAP had higher viscosity and elastic modulus, but HMAP-CD demonstrated superior performance. PHPA, HMAP, and HMAP-CD achieved final oil recovery of 50.0%, 68.8%, and 74.5% in porous medium experiments [152]. Incorporating cyclic groups into hydrophobically modified polyacrylamides is a viable way to improve their efficiency for EOR. Xie et al. studied modified polyacrylamide containing cyclodextrin groups [153]. Zhong et al. studied xanthan gum viscosity with varied ionic strengths [154]. Low polymer concentrations reduced viscosity with inorganic cations, particularly Ca^2+^ [155]. At elevated polymer concentrations, xanthan gum viscosity rose with increasing inorganic cation levels. Adding 200–1000 mg/L Ca^2+^ boosted viscosity by 475% at 5000 mg/L xanthan gum. The thermal stability of xanthan gum increases with the salinity of the aqueous solution. Xanthan gum exhibits non-Newtonian behavior often analyzed with mathematical models [154]. The polymer shows high viscosity at low shear rates due to hydrogen bonding and polymer entanglement. With increasing shear rates, viscosity decreases, indicating shear-thinning behavior. Guar gum’s high hydrating capacity results in elevated viscosity at low shear rates due to increased hydrodynamic volume and intermolecular interactions. With rising shear rates, it displays shear-thinning behavior [156]. Guar gum viscosity rises with solution salinity, but divalent cations induce polymer precipitation. Insoluble at low temperatures, its viscosity increases; however, it decreases at higher temperatures.

#### 3.2.2. Shear-Thinning Behavior

**Figure 12:**
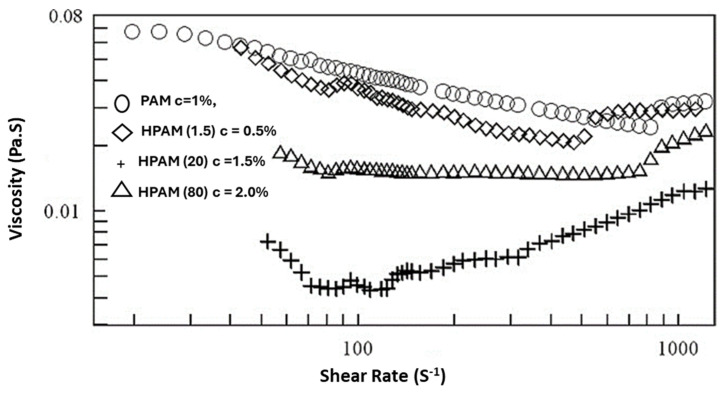
Plot of apparent shear viscosity versus shear rate for PAM and HPAM in aqueous/NaCl solution at 298 K [157].

Xanthan gum polymer chains disentangle and align beside the flow direction, giving rise to significant shear-thinning. Shear-thinning polymer solutions were developed for EOR drilling fluids to suspend cutting at low shear rates while allowing for easy flow at high shear rates. Shear-thinning can be compared to fluid’s pseudoplastic behavior in rheological investigations. Shear-thinning behavior refers to fluid activity that occurs under stress. Polymer solutions follow the power law equation because of their shear-thinning nature. The viscosity curves for solutions of PAM and HPAM in 1 and 2% aqueous NaCl are presented below in Figure 12.

PAM and HPAM samples exhibit non-Newtonian behavior, with shear-thinning below the critical shear rate and shear-thickening above it. In the first area (γ<γc), viscosity decreases with shear rate due to macromolecule orientation and disentanglement with increasing shear force. Shear-thickening may occur due to changes in macromolecule shape caused by flow, including deformation and interaction of macromolecules. Unfolded macromolecules may form transient networks or entanglements during flow conditions. In γ<γc, the unfolded polymer chains create a transient network, increasing the viscosity of the fluid. When shear stops, the network relaxes, returning chains to coil configuration. Shear forces can disrupt electrostatic charge screening in HPAM molecules, leading to an increase in **η***a* in the shear-thickening area due to negative charge repulsive forces. In HPAM samples with a high degree of hydrolysis (DH ~ 20%) and a high shear rate in the above (Figure 12), orientation and disentanglement effects are compensated for by deformation and association effects. HPAM (20) and HPAM (80) show Newtonian behavior at specific shear rates.

## 4. Crosslinking Mechanisms

**Figure 13:**
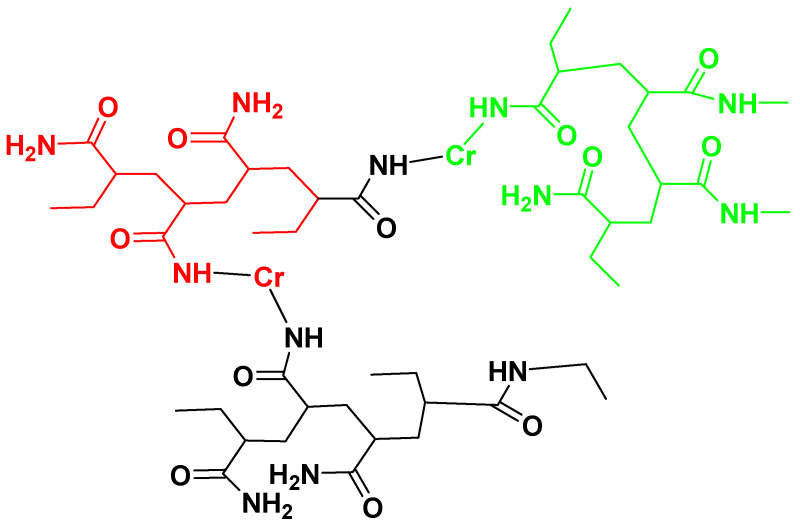
Chromium metal ions crosslink polyacrylamide, creating a complex networked gel [160].

Polymers composed of high-molecular-weight monomer chains elevate liquid viscosity through internal friction with surrounding solvent molecules due to their coiled structures. Polymer solution viscosity depends on type and solvent. Linear polymers form network structures via crosslinking, which varies by condensation, sulfur vulcanization, irradiation, or chemical interactions with metals under heat or pressure [158]. The crosslinking process alters the chemical structure of polymers independent of the mechanism used. Fracturing fluids to form gels typically requires a polymer concentration of 20–40 pptg [159]. A polyacrylamide polymer with carboxyl groups is crosslinked, resulting in a gel, as shown below in Figure 13 [160].

Crosslinking involves creating chemical or physical bonds between polymer chains, yielding a three-dimensional gel network, and profoundly influencing polymers’ physical and mechanical traits. The physical architecture of macromolecules, particularly polymers, can be altered by a variety of crosslinking mechanisms even though, with current progress in polymer chemistry, several methods can be utilized to prepare crosslinked gels; however, the two most common methods are chemical and physical crosslinking.

### 4.1. Chemical Crosslinking

**Figure 14:**
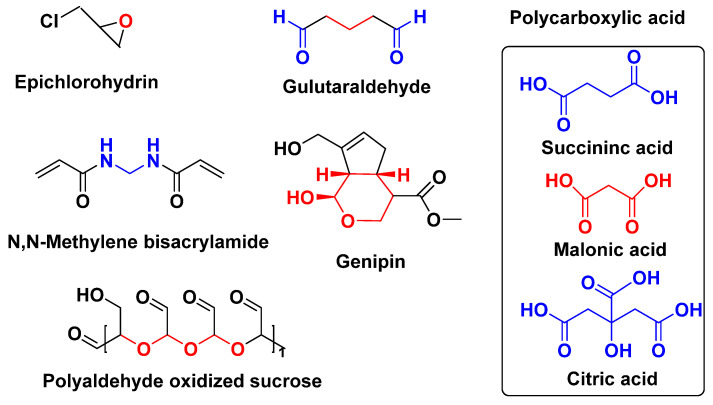
Chemical crosslinkers for starch-based SAPs.

High-energy irradiation induces chemical crosslinking without additional crosslinking agents. Γ-Irradiation generates macro radicals, causing polymer chain breakdown in the solid state and impacting material crosslinking in both the solid and solution states. Polymers respond to radiation dosage, temperature, molecular structure, and morphology. Γ-Irradiation in solution benefits from enhanced polymer chain mobility sustaining degradation resistance. Pre-irradiation solution degassing minimizes polymer degradation [161]. Gamma-ray irradiation induces free radical formation in starch depolymerizing amylose and amylopectin. When copolymers are present, starch-copolymer radicals crosslink to create superabsorbent polymers [162,163]. Incorporating natural or synthetic polymers enables specific modification of SAPs [164], and this is highlighted in Figure 14 below.

El-Mohdy et al. employed γ-irradiation to copolymerize acrylic acid with gelatinized maize starch in water [165]. Higher irradiation dosage and acrylic acid content increased gel content, reducing starch/acrylic acid SAP swelling. The SAPs exhibited greater thermal stability than starch and polyacrylic acid. Maximal water absorption rates differed. The starch/acrylic acid SAPs swelled lower in NaCl solution than in distilled water. The neutralized starch/acrylic acid SAPs showed high swelling, which is suitable for agricultural use.

SAPs with and without 1% MMT were synthesized via γ-radiation, with MBA being used as a crosslinker. Unfilled SAPs received varied doses (2.5–10 kGy), while clay-filled SAPs were irradiated at 2.5 kGy. Clay-filled SAPs exhibited higher swelling in water than saline solutions with reduced water retention as clay content increased [166]. When starch partially replaced carboxymethyl cellulose (CMC) the resultant SAP exhibited enhanced water swelling compared to pure CMC gels. Starch-enhanced gel fraction (55%) and water uptake (350 g/g) at 20 kGy were added, compared to pure CMC gels (35%, 200 g/g). The optimal SAP combined 30% starch and CMC, but excessive starch reduced gelation due to radiation degradation. When combined with CMC, starch radicals crosslink with CMC radicals rather than degrade. This results in a heterogeneous structure with dispersed starch granules and fragments showing less sensitivity to ionic strength [164]. Microwave irradiation enhances cornstarch-based SAP synthesis, yielding higher swelling ratios and lower solubility than conventional methods. Grafting sodium acrylate onto cornstarch with PPS as an initiator and polyethylene glycol diacrylate as a crosslinker achieves optimal results at 85–90 W power and 10 min irradiation. Swelling capacity reaches 520–620 g/g in distilled water [167].

Cassava starch underwent radiation grafting copolymerization with acrylamide using a 10 MeV electron beam initiator followed by MBA crosslinking and alkaline hydrolysis. The resulting composite had a maximum absorption capacity of 1452 g/g for distilled water and 83 g/g for saline solution. Optimal conditions included an 8 kGy total dose, a 4.5 mol/mol acrylamide-to-β-anhydroglucose ratio, and a 0.4% mol/mol crosslinker-to-acrylamide ratio. The study highlighted the superior efficiency of 10 MeV electron beam irradiation over γ-ray irradiation in promoting polymerization reactions [168].

### 4.2. Physical Crosslinking

**Figure 15:**
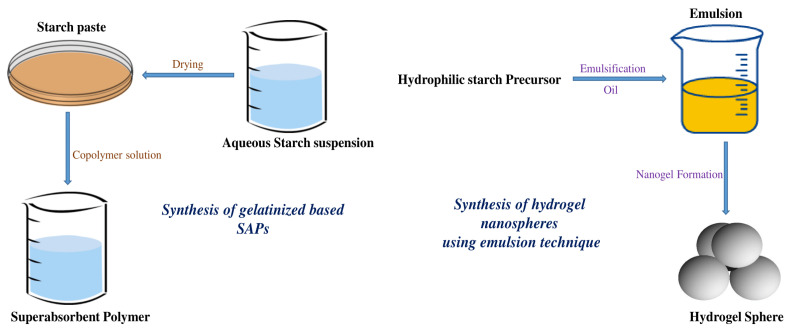
Physical crosslinking in starch-based superabsorbent polymers (SAPs) [173].

Pregelatinization of starch involves transforming an aqueous starch suspension into a paste through gelatinization at high temperatures. Pregelatinized starch is obtained by gelatinizing and drying the starch solution [169]. Heating starch in water to 55–85 °C changes its helices, making it soluble in cold water and causing significant swelling [170], while utilizing copolymer solution in pregelatinized starch can help in eco-friendly SAP creation [161]. SAP, or physically crosslinked starch, can be used to create nanogels by procedures such as reverse emulsification. Emulsification entails adding a hydrophilic starch precursor to a hydrophobic liquid, forming tiny droplets via agitation. Controlling agitation conditions can impact both gel size and nanogel characteristics [171]. The swelling ratios of the produced starch nanohydrogels ranged from 2.0 to 14.0 and were determined by amylose content. Pea starch nanogels have lower water absorption values than potato starch nanogels [172]. Others have also shown a method of preparing hydrogel spheres starting with starch using physical crosslinking methods, as described in Figure 15 below.

A modified twin-roll HAAKE mixer was used to produce high-viscosity materials including starch-based superabsorbent polymers (SAPs). These SAPs were coated with urea fertilizer, regulating its release in water [163,174]. In a separate investigation, a coated fertilizer system was developed using starch acetate (SA) and weakly crosslinked carboxymethyl starch/xanthan gum (CMS/XG) to enhance biomass utilization efficiency and water utilization efficiency, as well as to mitigate nutrient loss and environmental pollution. The dual-coated fertilizer exhibited slow-release properties, achieving nitrogen equilibrium release within 20 days. Controlling coating thickness and plasticizer quantity regulated nitrogen release kinetics in soil benefiting from polysaccharide-based coatings [175]. Singh et al. integrated kaolin and bentonite into starch−alginate beads, crosslinked with a CaCl_2_ solution, for thiram fungicide release control. The presence of kaolin and bentonite in the formulation reduced equilibrium swelling from 84% to 64% and 72%, respectively. The slow release of thiram was attributed to better adsorption on clay particles and demonstrated a non-Fickian diffusion mechanism, with significantly lower release amounts (6.9 and 6.3 mg) from beads containing 4 wt% of kaolin and bentonite compared to pure starch–alginate beads (10 mg) after 300 h [176]. Table 11 below shows the concentration, viscosity, shear rate, and adsorption capacity of polymers utilized in a variety of applications. XG requires concentrations of 0.10 wt%, 0.25 wt%, and 0.50 wt%, with viscosities ranging from 13 to 35 cPs and an adsorption capacity of 435 mg/g. HPAM versions (1.5), (20), and (80) at concentrations of 0.5, 1.5, and 2 wt%, respectively, display viscosities ranging from 9.0 to 20.2 cPs and adsorption capacities of 1.17–1.62 μg/cm^2^. At a 1 wt% concentration, PAM has a viscosity of 11.8 cPs and an adsorption capacity of 75 mg/g.

## 5. Effects of Polymer Interaction with Reservoir Conditions

### 5.1. Temperature Sensitivity

The temperature effect on the rheology of hydrophobic polyacrylamide is well documented [177,178,179]. The temperature sensitivity of HAPAM depends on concentration, notably in dilute and semi-dilute states. At concentrations below the critical association concentration (CAC), polymer viscosity diminishes with rising temperature due to weakened intermolecular associations and endothermic intramolecular processes [180]. Beyond CAC, viscosity rises then declines, marking semi-dilute thermal behavior. El-Hoshoudy et al. examined the thermal properties of a synthesized acrylamide-based HAPAM polymer at 2 g/L, 7.34/s and 25–100 °C [181]. They observed an increase in viscosity of up to 50 °C, followed by a decrease of up to 100 °C. This behavior is attributed to endothermic hydrophobic intermolecular associations forming a network/micro-domain of polymer chains, increasing hydrodynamic volume and viscosity. Beyond 50 °C, thermal-induced motion weakens the super-aggregate structure, reducing intermolecular association and polymer viscosity. Similar trends were reported by [182,183] for various HAPAM polymers, indicating the influence of hydrophobic co-monomers on temperature-dependent viscosity and emphasizing the significance of co-monomer type, quantity, and molecular composition in HAPAM polymer synthesis. Table 12, as described by Afolabi et al., outlines the peak temperature strength of chosen HAPAM polymers at semi-dilute concentrations. Hydrophobic comonomer integration boosts thermal resilience [184]. However, variability in polymerization activity can yield HAPAM polymers with lower intrinsic viscosities and molecular weights, leading to disparate temperature tolerances [185]. Consequently, thermo-thinning defects manifest beyond temperatures associated with maximum viscosity. The molar ratio of hydrophobic comonomers (10–30%) influences polymer performance, with higher ratios enhancing efficacy but increasing production costs. Andrades Equation (1) applies to hydrophobic polymers, with activation energy closely resembling energy barrier comparisons.

(1) $$\eta =Be\left(\frac{Ea}{kT}\right)$$

In simpler terms, a transition from hydrophilic to hydrophobic behavior occurs in HAPAM polymers, as indicated by Li and coworkers [152]. The critical aggregation temperature (CAT) is determined by the critical solution temperature of the graft monomer, often N-isopropylacrylamide (NIPA) [125,189,190,191]. LCST signifies the temperature at which components in a mixture are soluble, while some monomers show an upper critical solution temperature (UCST) [189]. Fine-tuning polymer viscosity aids high-temperature oil reservoir usage. The critical solution temperature of grafted polymers varies based on factors like polymerization degree, branching, and polydispersity. This may result in low molecular weight requiring increased polymer concentrations for thermo-thickening.

### 5.2. Salinity

The saline character of reservoir conditions makes polymer gel performance in oil and gas operations difficult. High quantities of inorganic salts can substantially impact the gel structure. Cations in a high-salinity environment cause a decrease in electric potential on the polymer surface, resulting in molecular structural contraction and decreased swelling performance [192]. In situ, crosslinked gels can improve salt resistance by modifying resorcinol, HMTA, and oxalic acid [193]. Furthermore, surface crosslinked polymer gels and calcium alginate gels with nano clay show increased stability under high-salt circumstances. Non-crosslinked polymer gels that rely on hydrogen bonds and intermolecular forces demonstrate good salt resistance, further enhanced by ion−ion interactions, with ionic liquids providing a viable pathway for successful performance in various reservoir salinity ranges [194,195]. Various methods exist to address high salinity challenges in HAPAM polymers, such as introducing salt into a prepared polymer solution. The method used to prepare HAPAM polymers in water and brine affects their rheological properties [196,197]. Maia et al. used three methods to study how monovalent ions (Na^+^) affect an acrylamide-N, N-dihexylacrylamide copolymer. The copolymer’s viscosity decreased in the salt solution due to cation screening. Adding salt powder led to increased viscosity, which was attributed to surfactant presence, while adding saltwater increased viscosity, which was attributed to ease of interaction and the formation of a polymer chain network [196]. Mansha et al. synthesized properties of exceptionally thermo-switchable viscoelastic responsive zwitterionic gemini surfactants in highly saline water [198]. Al-Sabagh et al. observed that divalent ions (Ca^2+^) affected HAPAM polymers differently than monovalent ions with a limited understanding of the associated phenomenon [177]. The concentration regime (dilute or semi-dilute) affected salt tolerance, influencing the salt-thickening capability of associating polymers with brine. Beyond a certain ion concentration, polymer viscosity decreased due to a salting-out effect, potentially causing polymer precipitation. Crosslinking polymer chains have also been explored to enhance the salt-thickening capabilities of high-permeability reservoirs [185]. Ali et al. noted that temperature impacts xanthan gum and HPAM polymers, while xanthan gum exhibits superior stability under harsh conditions [199].

### 5.3. Effect of High Pressure

High-pressure interactions between polymers and reservoir conditions can have significant effects on the performance and effectiveness of hydraulic fracturing operations. High pressure can alter the viscosity of polymer solutions. Under high pressures, polymer chains may become more entangled and compressed, increasing viscosity. This change in viscosity can affect the flow behavior of fracturing fluids, limiting their ability to properly transport proppants and penetrate cracks [184]. In high-pressure conditions, polymer-based fluid loss control additives may perform better. Increased pressure can improve polymer adsorption on the formation matrix, lowering the loss of fluid into the reservoir rock. This action is especially important for keeping hydraulic fractures intact and reducing formation damage [200]. High pressure can influence the opening and maintenance of hydraulic fractures caused by fracturing procedures. Polymer interactions with reservoir conditions, particularly pressure, can influence fracture width and conductivity. To promote long-term fracture conductivity and maximum hydrocarbon recovery, proper polymer selection and fracturing fluid composition optimization are required.

## 6. Macromolecular-Based Fracturing Fluids—The Challenges

### 6.1. Environmental Concern

Concerns about the environment in macromolecular fracturing fluids stem from the retention and breakdown of polymeric gels in formations that introduce toxicity and chemical pollutants into the reservoir via leakage of oligomeric and monomeric units from the breakdown of the polymeric gels. Traditional crosslinkers, which are frequently hazardous chemical reagents, can pose particular problems as far as environmental impact is concerned due to the phenomenon of incomplete or partial in situ gelation. While zirconium acetate-based gels are salt and shear-resistant, problems nevertheless arise during migration, creating low-permeability layer pollution and impeding recovery. Although innovative technologies such as degradable gel particles and low-elastic polymer gel microspheres strive to lessen environmental effects, obstacles remain in regard to attaining maximum environmental protection without reducing gel characteristics. Balancing higher expenses with improved environmental performance is still an important aspect of tackling these difficulties [60,201].

### 6.2. Degradation

Using enzymes for polymer degradation in hydraulic fracturing poses certain challenges due to extreme reservoir conditions. Enzymes are sensitive to factors like temperature, pressure, salt concentrations, and pH. Advances in proteomics and molecular biology, along with isolation strategies, have addressed these challenges to some degree. Thermophilic enzymes, capable of polymer degradation at high temperatures, have been successfully employed in hydraulically fractured reservoirs at temperatures up to 150 °C [202]. Studies demonstrate enzymatic activity across diverse salinities and pH ranges, enhancing the applicability of enzymatic polymer degradation in challenging oil reservoir environments. Cobianco et al. found amylase and glucanase enzymes remained active, degrading starch and xanthan gum in diverse brines [203]. A challenge in using enzyme breakers is controlling break duration and enzyme stability until the polymer reaction. Enzyme breakers are typically introduced post-proppant delivery into fracturing fluids. Recent techniques tackle reaction times and stability challenges. Chopade et al. discovered lignosulfonates stabilized guar gum-degrading enzyme activity at high temperatures and pH levels [204]. Barati et al. proposed a nanoparticle system to stabilize enzyme breakers in alkaline, high-temperature conditions [205].

**Figure 16:**
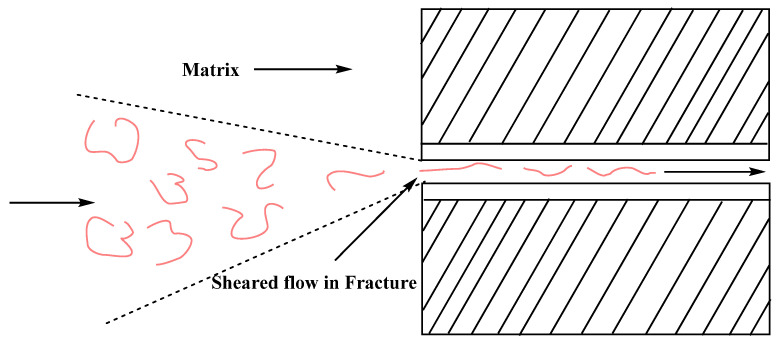
Shear-induced mechanical degradation of polymers in fractured reservoir flow [130].

High shear rates and elongation deformations cause mechanical degradation in polymers, breaking molecules. In the context of oil and gas applications, Figure 16 depicts the occurrence of mechanical degradation caused by high shear rates during turbulent flow through fractures [130]; it involves irreversible polymer molecule breakdown highlighting mechanical stress challenges [206].

High shear rates causing mechanical degradation in polymers lead to the unwinding of polymer chains, significantly reducing viscosity and impairing displacement efficiency and drag reduction capabilities. The choice of polymer for fracturing operations is intricately tied to rheology, with shear-thickening behaviors posing challenges such as wellbore fracturing or mechanical degradation. Synthetic polymers, particularly polyethylene oxide, and polyacrylamide, initially exhibit superior drag reduction. However, their efficiency diminishes faster under shear compared to the more shear-stable xanthan and guar gum, making the latter more suitable for high-flow-rate applications [207,208]. Mixing synthetic polymers with xanthan/guar gum is explored as a potential strategy to enhance the mechanical degradation resistance of synthetic polymers [209]. Polymer mechanical degradation intensifies at elevated flow rates and lower permeabilities, particularly in slick-water fracturing applications for low- and ultralow-permeability reservoirs, such as shale reservoirs. The stress on the polymer in these conditions leads to chain breakage, causing a significant reduction in fluid viscosity.

**Figure 17:**
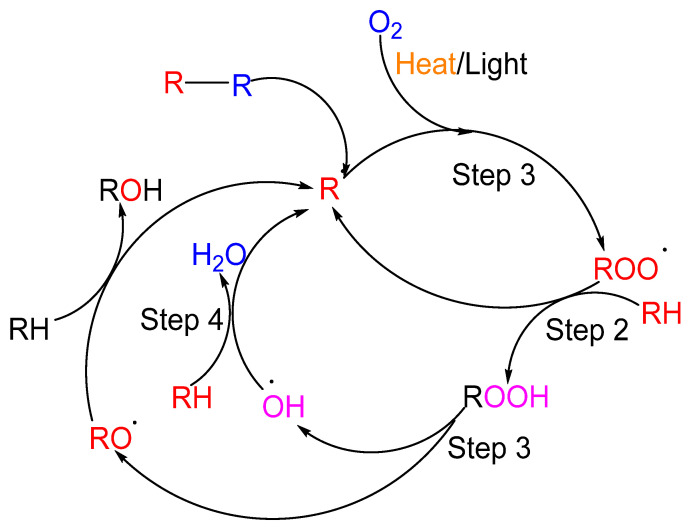
Polymer thermal degradation mechanism [210].

In the oil and gas industry, extensive efforts have been directed toward enhancing the thermal properties of polymers for applications in EOR and the fracturing of high-temperature unconventional reservoirs. Guar gum lacks stability beyond 350 °F, and research investigates synthetic polymers like AMPS-acrylamide terpolymer that endure over 450 °F. Thermal degradation, a consequence of polymer overheating, alters their properties, leading to the breakdown of polymer molecules. This process involves initiation, propagation, and termination steps, as illustrated in Figure 17 [210].

### 6.3. Challenging Reservoir Conditions

**Figure 18:**
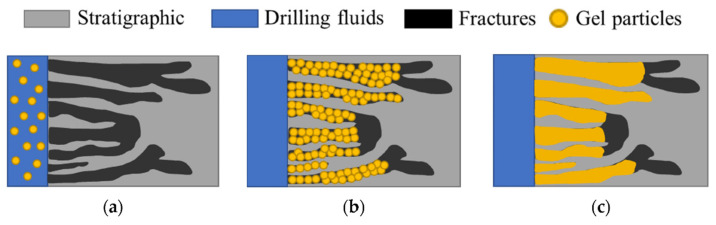
The process of polymer self-healing gel plugging involves (**a**) circulation of gel particles in the wellbore with drilling fluid, (**b**) adsorption and accumulation of gel particles in formation fractures, and (**c**) gel particles extruded and deformed to seal fractures [212].

When faced with deep and complex reservoirs (heterogenous formations) that need particular or specialized construction work, the oil and gas industry needs to use novel techniques in engineering (fracturing) and careful selection of oilfield chemistry in conjunction with suitable drilling fluids. Moreover, the range of choices in applying particular engineering or drilling fluid applications is limited due to concerns regarding environmental protection. Therefore, industry expectations for novel drilling fluids envisage drilling fluids that are resistant to high temperatures, can cope with varying salinity, and can navigate heterogeneous formations smoothly to enhance the recovery of hydrocarbons. The two primary methods that prevent drilling fluids from seeping into clay formations are electrostatic interaction and intermolecular bonding (mostly hydrogen bonding). Because the majority of clay formations possess a negative surface charge, this encourages drilling fluid macromolecular additives with a positive charge to deposit onto the formation surface. To prevent drilling fluid polymer loss, it is imperative that after careful analysis of reservoir formation clays, suitable polymers of certain chemistry and charge are employed as additives in the drilling fluid. With this goal in mind, research and development efforts need to focus on preparing drilling fluids that can cope with reservoirs of varying temperature, pH, salinity, and heterogenous formations. Currently, a pressing issue for petroleum engineers is coping with well leakages in reservoirs, which, due to being deep, pose a high-temperature challenge. Moreover, the macromolecular gels that are being used as plugging material, suffer from poor mechanical properties, and they are are also not suitable for all reservoir formation types. This creates conditions whereby the macromolecular gels seal up the fractures in the formations. Whilst the option of employing pre-crosslinking gels is a possibility, it is unfortunately not a viable option for large fractures. In such cases, the crosslinked gel is prepared via extensive hydrogen bonding that leads to enhanced mechanical properties and facilitates reversible ionic bond formation via electrostatic interaction [211]. Polymer chains interconnect through physical entanglement, bolstering three-dimensional structure strength. After drilling gel particles adhere to formation fractures via hydrogen bonding and electrostatic forces, accumulation under formation pressure leads to self-healing upon water absorption, forming mechanically robust blocks (as shown below in Figure 18). Enhancing formation pressure resistance leads to sustained fracture-sealing effectiveness.

## 7. Prospects and Innovations

Polymer science progresses with innovations and trends impacting materials and technologies. Recent advances span stimuli-responsive polymers, smart materials, and sustainable production. Emerging trends promise solutions for oil and gas recovery challenges, reflecting evolving needs in polymer utilization within the industry.

### 7.1. Polymers That Respond to Stimuli: An Emerging Field

Because of their ability to respond to environmental cues, stimuli-responsive polymers, commonly called smart polymers or intelligent materials, have gained attention in polymer science. These polymers respond to a range of environmental stimuli, including temperature, pH, light, and electric fields, by changing their form, size, or solubility. Shape memory polymers are a prominent subclass of stimuli-responsive polymers (SMPs). SMPs are remarkably adept at remembering a particular shape and transforming back into a distorted condition in response to an outside stimulus. Applications for this trait can be found in engineering, aeronautical, and medical devices. SMPs are utilized for example in medical technology to create minimally invasive surgical instruments that, when heated, change shape to enable complex procedures to be performed with fewer incisions. Most often, pH-sensitive hydrogels have the potential to release pharmaceuticals based on changes in body pH, thereby enhancing drug delivery precision, improving therapeutic efficacy, and reducing side effects. Furthermore, these hydrogels contribute to the development of self-healing materials responding to pH variations, prolonging material life across diverse applications while stimuli-responsive polymers offer innovative solutions in environmental research and biotechnology, presenting opportunities for targeted pollutant removal and advanced diagnostics [213].

### 7.2. Hybrid Materials: Moving beyond Polymers Alone

Recent advancements in smart materials extend beyond polymers, encompassing diverse materials that are responsive to external stimuli. In structural engineering, the fusion of shape memory alloys (SMAs) and polymers yields self-monitoring and repairing materials, which are particularly beneficial in constructing adaptive buildings and bridges. In electronics, conductive polymers like poly (3,4-ethylenedioxythiophene) contribute to flexible and stretchable devices, enabling wearable technology, bendable displays, and soft robotics. Further innovations involve nanomaterials such as carbon nanotubes and graphene, enhancing polymer properties for applications in sensors, nanoelectronics, and advanced composites [214]. With this in mind, it is highly probable that, shortly, we will see innovations in material science and engineering move beyond just simple polymer solutions and proppants in the area of fracturing fluids. A new generation of fracturing fluids will probably contain polymers that are incorporated on the surface of nano or microparticles via covalent linkages or through being physisorbed. This suggests a hybrid system that incorporates a polymeric organic layer on a dense inorganic particle, thereby giving rise to novel materials that can be fine-tuned to possess certain physical and mechanical properties, as opposed to simply considering the chemical properties of traditional polymers as the proppant carrier, as is the case with current fracturing fluid additives.

### 7.3. Sustainable Polymer Production: A Greener Future

Sustainability drives modern polymer chemistry concerns over petrochemical-based polymers’ environmental impacts, including plastic pollution and resource depletion. Sustainable polymer manufacturing is becoming a key area of study and innovation. The creation of bioplastics is one method for achieving sustainable polymer production. Polymers sourced from renewable origins, like plant starch, algae, or agricultural waste, are gaining traction. Microbial synthesis yields biodegradable polyhydroxyalkanoates, offering eco-friendly alternatives to conventional plastics. Plastic recycling, upcycling, and bioplastic adoption are rising trends in sustainable polymer usage. Scientists are converting waste plastics into valuable resources via depolymerization and chemical recycling, reducing environmental impacts and bolstering recycling economies. Bio-based monomers and green chemical approaches contribute to the development of sustainable polymers. Monomers derived from biomass can form diverse polymers like polyesters and polyamides. Green chemistry principles aim to minimize hazardous compounds and energy usage in polymer synthesis, modifying environmental impacts during manufacturing [215].

## 8. Conclusions

The study of macromolecules for fracturing fluid applications in the oil and gas industry not only highlights the increase in a variety of promising materials as efficient fracturing fluids for water-based fluids, but also provides an opportunity to reassess where research and development efforts can move to increase efficiency, reduce cost of operation, and introduce more environmentally friendly systems for oil and gas recovery. In polymer chemistry, one particular focus that has recently gained attention is the use of synthetic hyperbranched polymers with various chemical structures and features that may provide additional advantages. The interaction of these macromolecules with reservoir conditions has been examined, and details regarding their performance have been provided. Degradation scalability and environmental issues are two major challenges requiring careful optimization. Prospects depend on addressing these difficulties through new ideas and environmentally responsible alternatives. Future research endeavors should focus on developing novel macromolecular architectures and formulations to improve fracturing fluid efficiency, and hybrid materials will likely emerge that encompass the best features of organic polymers and inorganic particles to meet the challenges of preparing fracturing fluids with superior efficiencies. If ideal fracturing fluid formulation conditions can be met, then implications for the oil and gas industry will lead to the possibility of increased well stimulation and production. This comprehensive understanding provides the foundation for ongoing innovation in macromolecular chemistry in fracturing fluids, focusing on addressing industry-specific challenges.

## Figures and Tables

**Table 1:** Type of polymer and its application in hydraulic fracturing [23].

Types of Polymers	Gelling Agent	Use	Ref
Guar-based	Guar gum Hydro propyl guar Carboxymethyl guar Carboxymethylhydropropyl guar	Proppant delivery agent Fluid loss additive Gravel packing Thickener	[24]
Cellulose-based	Hydroxyethyl cellulose Hydroxypropyl cellulose Carboxymethyl cellulose Carboxymethylhydroxyethyl cellulose Methyl cellulose	Proppant delivery agent Fluid loss additive Gravel packing Thickener	[24]
Acrylamide and acrylic acid-based	Polyacrylamide Polyacrylate Methyl acrylamide Acrylic acid Methyl acrylic acid Copolymers from acrylamide and acrylic acid	Friction reducer Proppant delivery agent Fluid loss additive Gravel packing Thickener	[24,25]
Starch-based	Xanthan gum Starch and its derivatives Polyurethanes, Polyesters	Proppant delivery agent Foaming agent Gravel packing Drilling muds Thickener Fluid-loss additive	[24,26]

**Table 2:** Common polymers that are derived from guar gum [30].

Derivative	Structure of Substituent	Ionic Charge
Hydroxy Propyl Guar (HPG)	CH_2_-CH(OH)CH_3_	Nonionic
Carboxy Methyl Guar (CMG)	CH_2_-COO-Na^+^	Anionic
Carboxy Methyl Hydroxy Propyl Guar (CMHPG)	CH_2_-COO-Na^+^ CH_2_-CH(OH)CH_3_	Anionic

**Table 3:** Application of guar gum and its derivatives in corrosion inhibition, fracturing fluids, enhanced oil recovery, and viscosity improvers.

Name of Polymer	Concentrations Applied/Used (wt%/mol/mmol)	Efficiency of Corrosion Inhibition (%)	EOR (%)	Viscosity (cPs)	Modifier/Activator	Ref.
Guar Gum (GG)	0.1–0.5 wt%	85%	60%	500–1000	Borax	[30]
Hydroxypropyl Guar (HPG)	0.2–0.8 wt%	90%	65%	600–1200	Calcium chloride	[35]
Carboxymethyl Guar (CMG)	0.1–0.6 wt%	80%	55%	400–900	Sodium hydroxide	[30]
Carboxymethyl Hydroxypropyl Guar (CMHPG)	0.3–1.0 wt%	95%	70%	700–1500	Zinc chloride	[35]

**Table 4:** Drilling fluid additives containing cellulose and its derivatives [64].

Material Used	Property of Material Studied	Major Findings	Ref
CMC	Clay has hydration-inhibiting characteristics.	During 180 min, the clay swelling degree was marginally lower in the low molecular weight CMC than in the high molecular weight CNC.	[65]
CMS	Properties of Rheology	The combined action of CMC and PVP enhanced the apparent viscosity and the shear stress, the tackifying effect of CMC was greater than that of PVP.	[66]
CMC	Rheological properties, filtration, and interfacial properties	The combined action of CMC and PVP enhanced the apparent viscosity and the shear stress; the tackifying effect of CMC was greater than that of PVP.	[59]
PAC	Volume of filtrate and viscosity	Drilling fluid’s viscosity can be efficiently increased, and its filtrate volume decreased, with PAC.	[67]
PAC	Rheological properties	It would appear appropriate to treat PAC solutions as merely viscous, shear-thinning for cuttings transport simulation.	[68]
HEC	Properties of Rheology	Drilling fluid with optimal rheological characteristics can be guaranteed by incorporating HEC at the optimal concentration into bentonite suspension.	[62]

**Table 5:** Various starch derivatives are utilized in EOR applications [75].

Modification	Improvement	Oil Recovery Factor	Reservoir Type	Reservoir Conditions (T, Salinity, Porosity and Permeability)	Ref
Acryloylated starch-gpoly (AM-AA)/TMVS	Expand the water absorption capability of polymers and enhance their salt tolerance.	46% of S_or_	Sandstone	70 °C, 80,000 ppm, 21.9% and 45.39 mD	[71]
Acryloylated starch-gpoly (AM-VMA-NVP)/ DMPVS	Enhance thermal stability and salt tolerance (severe reservoir conditions).	49% of S_or_	Sandstone	80 °C, 80,000 ppm, 21.9% and 45 mD	[73]
Sulfonic starch-g- MBAM&TEVS	Boost wettability to promote hydrophilic characteristics.	39% of S_or_	Sandstone	50 °C, 100,000 ppm, 22% and 45.4 mD	[72]
Crystalline starch nanoparticles (CSNP)	Reduce interfacial tension (IFT) and enhance the wettability of the rocks to promote hydrophilic properties.	23% of OOIP	Sandstone	120 °C, 22,000 ppm, 15.3% and 167.43 mD	[79]
Quaternary ammonium cationic starch	Enhance wettability to raise hydrophilicity and increase salt tolerance.	18% of OOIP	Sandstone	70 °C, 5727 ppm, 25% and 1.46 μm^2^	[80]

**Table 6:** Comparison of natural polymers over synthetic polymers.

Aspect	Natural Polymers	Synthetic Polymers	Ref
Biodegradability, Biocompatibility, Non-toxicity	Naturally occurring polymers are biodegradable, biocompatible, and non-toxic. Plant-derived materials are composed of complex carbohydrates, rendering them safer than synthetic counterparts synthesized from potentially harmful chemicals.	Synthetic polymers are often derived from chemical synthesis, which may introduce toxins and pollutants. Depending on their chemical composition, they can have varying levels of biodegradability and biocompatibility.	[87]
Economic Viability	Natural polymers typically have lower production costs compared to synthetic polymers. They are sourced from readily available plant and animal materials, leading to cost-effectiveness.	Synthetic polymers may involve complex manufacturing processes and the use of petrochemicals, which can contribute to higher production costs. However, economies of scale and technological advancements may offset some of these costs.	[88]
Safety and Side Effects	Naturally occurring polymers are generally considered safe and devoid of side effects. They do not pose risks of chemical toxicity, making them suitable for various applications.	Synthetic polymers synthesized from chemicals may carry risks of toxicity and side effects, especially if they contain harmful additives or by-products. Proper regulation and testing are essential to ensure safety.	[86]
Availability	Natural polymers are widely available globally in animals, plants, fruits, and seeds. They are also used as food sources, contributing to their abundant supply. Industrial production ensures consistent availability.	Synthetic polymers may face supply constraints due to dependency on petrochemical feedstocks and manufacturing processes. Market dynamics and geopolitical factors can influence availability.	[88]

**Table 7:** The merits and drawbacks of various polyols derived from diverse sources.

Polyol Type	Advantages	Disadvantages
Polyether polyols are synthesized using propylene oxide and ethylene oxide	Hydrolytic resistance, economic viability, rheological properties, and flexibility.	Oxidative resistance, modulus/tensile strength, thermal stability, and flammability characteristics.
Aliphatic polyester polyol Aromatic polyester polyol	Oxidative resistance, modulus/tensile strength Flame retardancy, and modulus/stiffness.	Viscosity, hydrolytic resistance, Viscosity, and reduced flexibility.

**Table 8:** Synthetic polymers and their derivatives utilized as viscosity enhancers, increased oil recovery, fracturing fluids, and corrosion inhibitors.

Type of Polymer	Concentrations Applied/Used (wt%/mol/mmol)	Efficiency of Corrosion Inhibition (%)	EOR (%)	Viscosity (cPs)	Modifier/Activator	Ref.
Polyurethane	0.5–2.0 (wt% mol/mmol)	90%	60%	500–1500	Boron compounds	[22]
Polyamide	0.3–1.5 (wt%/mol/mmol)	85%	55%	400–1200	Phosphorus compounds	[22]

**Table 9:** Advantages and disadvantages of diverse synthesis methodologies.

Synthetic Method	Advantages	Disadvantages
Condensation Polymerization	High reaction efficiency and a wide range of monomer sources.	Synthetic polymers are easily hydrolyzed and multiple reaction steps.
Ring-opening Polymerization	Easy modification of polymer terminal groups and few reaction steps.	Limited sources of monomers.
Free radical Polymerization	A varied range of monomer sources, mild reaction conditions, and few reactions.	A wide distribution of molecular weights for synthetic polymers.

**Table 10:** Hyperbranched polymers and derivatives applied in petroleum industry functions.

Name of Polymer	Synthesis Method	Concentrations Applied/Used (wt%/mol/mmol)	Efficiency of Corrosion Inhibition (%)	EOR (%)	Viscosity (cPs)	Modifier/Activator	Ref.
Polymer A	Condensation	1–5 wt%	85%	60	1000	Sodium dodecyl sulfate	[125]
Polymer B	Radical	2–10 mol%	90	70	2000	Phosphonic acid	[126]
Polymer C	Ring-Opening	0.5–2 mmol/mmol	75	50	1500	Ammonium chloride	[125]

**Table 11:** Characteristics of particular polymer samples.

Type of Polymer	Concentration or Amount Needed	Viscosity	Shear Rate	Adsorption Capacity	Ref
XG	0.10 wt%, 0.25 wt%, 0.50 wt%	13–35	(0–1000 s^−1^)	435 mg/g (1.35 mmol/g)	[147]
HPAM (1.5) HPAM (20) HPAM (80)	0.5 wt% 1.5 wt% 2 wt%	20.2 12.4 9.0	500 380 700	1.17–1.62 μg/cm^2^	[157]
PAM	1 wt%	11.8	800	75 mg/g	[157]

**Table 12:** Maximum temperature resistance of certain HAPAM polymers examined at 170/s shear rate, 20–140 °C range [184].

Polymer	Maximum Temperature	CAC (g/L)	Concentration Regime	Ref
AM-AA-NIMA ^a^	42	0.80	Semi-dilute	[186]
AM-AMC12S-DPP ^b^	35	0.20	Semi-dilute	[187]
AM-DOAC-SSS ^c^	80	1.65	Semi-dilute (0.3 wt%)	[188]

**^a^** AM-AA-NIMA is a hydrophobically associating polyacrylamide (HAPAM) composed of NIMA and AA monomers. **^b^** AM-AMC12S-DPP is a hydrophobically associating polyacrylamide (HAPAM) polymer consisting of 1-(4-dodecyloxy-phenyl)-propenone (DPP) and 2-(acrylamido)-dodecanesulfonic acid (AMC12S) monomers. **^c^** AM-DOAC-SSS is a HAPAM comprising N, N-dimethyloctadeyl allyl ammonium chloride (DOAC), and sodium 4-styrenesulfonate (SSS) monomers.
